# Addressing Health Disparities Across the Cancer Continuum—a Los Angeles Approach to Achieving Equity

**DOI:** 10.3389/fonc.2022.912832

**Published:** 2022-07-05

**Authors:** Laurel J. Finster, Celina H. Shirazipour, Loraine A. Escobedo, Myles Cockburn, Zul Surani, Robert W. Haile

**Affiliations:** ^1^Cancer Research Center for Health Equity, Division of Medical Oncology, Cedars-Sinai Medical Center, Los Angeles, CA, United States; ^2^Norris Comprehensive Cancer Center, University of Southern California, Keck School of Medicine, Los Angeles, CA, United States

**Keywords:** cancer prevention and control, health equity (MeSH), healthcare disparities (MeSH), cultural diversity, social determinants of health (MeSH), community outreach and engagement

## Abstract

**Introduction:**

Different models have been developed to address inequities across the cancer care continuum. However, there remains a scarcity of best practices on understanding and responding to the burden of cancer in a defined catchment area.As such, the National Cancer Institute (NCI) recently provided a framework to maximize the impact on cancer burden, including a greater focus on community outreach and engagement. In this paper, we describe how Cedars Sinai Cancer (CSC), a health system that serves one of the most diverse counties in the US, implemented the framework to define its catchment area, characterize its population, identify high risk priority groups, and make decisions to address health disparities.

**Methods:**

We provide a review of the methods used to assess socio-ecological levels of influence. Data were reviewed from numerous national, statewide, and county sources and supplemented by locally administered questionnaires, heat maps, and community profile summaries to gain more localized snapshots of cancer disparities in Los Angeles County. Lastly, feedback was solicited from external peer groups, community stakeholders, and key decision-makers, and the proposed catchment area was aligned with the State’s Cancer Plan and the NCI Catchment Area and Community Outreach and Engagement Mandate.

**Results:**

The selected CSC catchment area meets NCI criteria and has potential to demonstrate impact both at the population level and within specialty populations. As a result, strategies are being developed to organize community outreach and engagement, as well as research across basic, clinical, and population sciences to guide cancer control and prevention efforts.

**Discussion:**

To maintain a high level of cultural inclusion and sensitivity, multiple layers of data are needed to understand localized pictures of cancer disparities and underlying causes. Community engagement remains essential to implementing policy, best practice, and translational science for broader impact.

**Impact:**

The clinical and translation work conducted at any cancer center requires an understanding of the determinants of health that contribute to the differences in cancer incidence and mortality among different groups. The NCI-aligned approach that we highlight is critical to support the design of future cancer control strategies that address and possibly reduce local health inequities.

## Introduction

Health disparities exist based on social, economic, and environmental factors, including gender, race, ethnicity, gender identity, sexual orientation, age, disability, geographic location, and socioeconomic status (1). Many different models have been developed to suggest how to address these disparities (2–6). What all models have in common is the intersection of multiple health domains (e.g., health behaviors, the built environment, health systems, etc.) and socio-ecological levels of influence (e.g., individual, interpersonal, community, and social levels) (7–10).

The National Cancer Institute (NCI) now requires cancer centers to define their catchment area with geographical boundaries, and address cancer burden and inequities within that region through research and community outreach and engagement (11). To support this goal, the NCI outlined seven areas for research and outreach activities (12): (1) define the catchment area (i.e. select the area and describe the demographics, special populations, and cancer burden); (2) assess the needs of the catchment area (i.e., basic, clinical, and population science research is conducted to address the cancer burden from prevention through survivorship); (3) engage the population in the catchment area (i.e., involvement of the population in setting a research agenda, and reaching out to the population through research, outreach, and education); (4) address disparities (i.e. identify and aim to develop solutions that decrease disparities for the populations experiencing cancer burden in the catchment area); (5) ensure that the demographics of the catchment area are represented in clinical trials (i.e. research studies reflect the demographic distribution of the chosen area); (6) translate research into policy (i.e. research should lead to policy change from local through international levels, including health care systems and government legislation); and (7) extend the reach of research and policy beyond the catchment area (i.e. collaboration with other cancer centers, organizations, and government).

The structure provided by these guidelines is essential when considering the vast diversity of municipal regions of the United States, such as Los Angeles, California, which is home to roughly 10 million people (13). The County has a large Latinx^1^ population, is considered the capital of Asia America, has the second-largest sexual and gender minority population in the country, spans a vast socioeconomic gradient, and covers both urban and semirural geographies (14–16). As such, the County is home to a large number of individuals who experience health inequities, with greater vulnerability among those who are foreign-born, lower socio-economic status, and living in areas with high ethnic concentration. Using a mixed methods approach, Cedars-Sinai Cancer embarked on a two-year assessment to meet NCI catchment area criteria while also maintaining a high level of cultural inclusion and sensitivity needed for serving one of the most diverse counties in the US.

## Methods

Below we highlight the series of steps taken to define, characterize, respond to, and engage the population in our cancer center catchment area.

### Step I: Defining the Catchment Area

**Figure 1:**
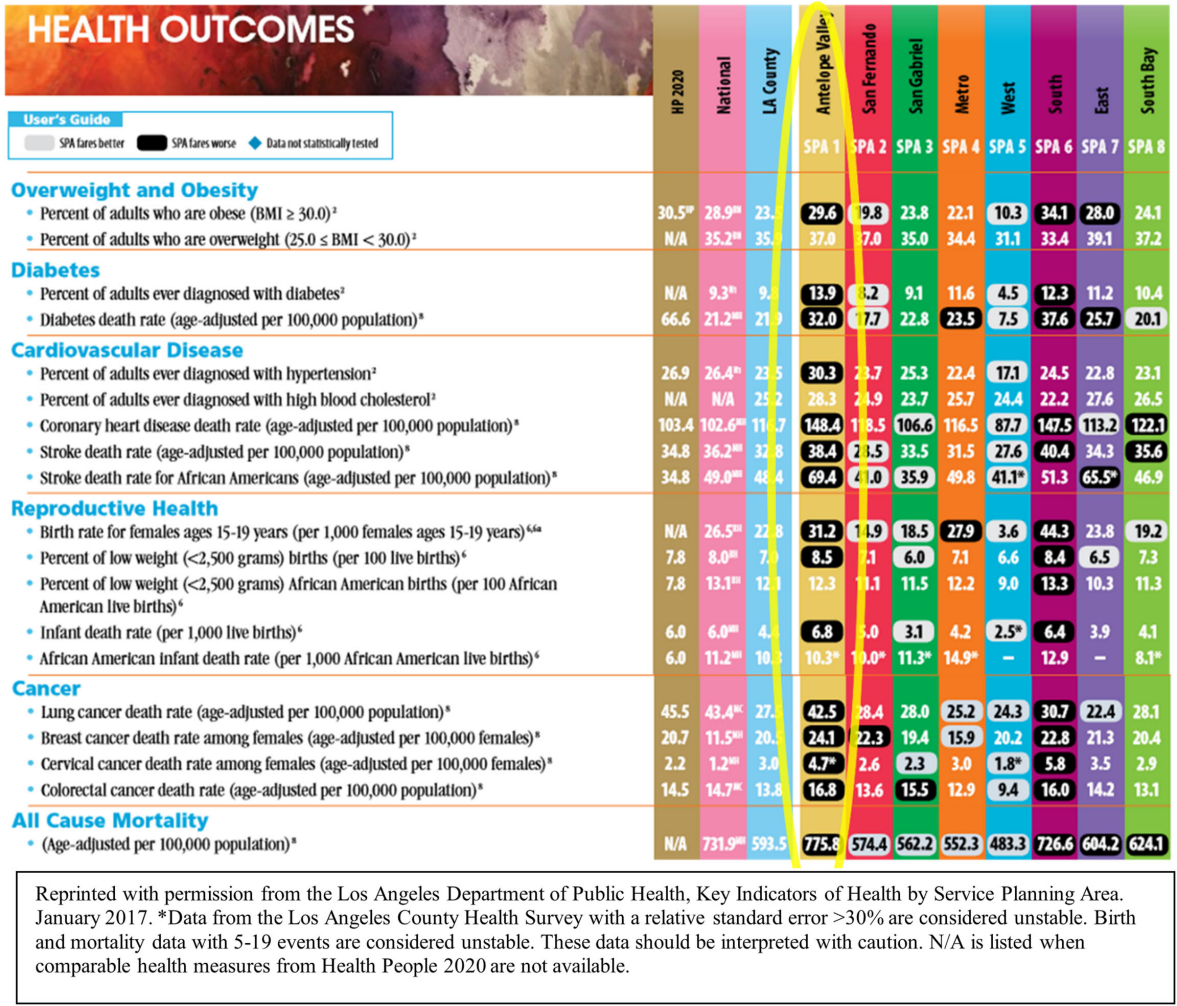
Los Angeles Department of Public Health, Key Indicators of Health by Service Planning Area.

Decisions were made regarding the catchment area based on geographic considerations, peer review to meet NCI criteria, and having a population size for which we could feasibly demonstrate measurable impact of our community outreach and engagement (COE) activities and COE-facilitated research. Our COE efforts focus on adherence to cancer screening guidelines and major behavioral and lifestyle factors, such as physical activity and tobacco use, and dissemination of the latest, most accurate cancer information. When considering the geographic area, we followed NCI metrics and County data. NCI requires clear geographic boundaries; a population of >4,000,000; greater than 80% of cancer patients residing within catchment area; and that the area is within 60 miles of the medical facility (CSC) to maximize clinical impact (12). These metrics were examined and linked with Los Angeles County (LAC) data on Service Planning Areas (SPAs).SPAs are geographic regions within LAC organized by the Department of Public Health. For each SPA, the county provides public health services, clinical services, and data targeted to the specific health needs of SPA-specific populations (17). Access to these data for smaller regions reveals important disparities that are often overlooked in aggregate data. For example, as noted in **Figure 1**, by breaking down key health indicators by SPA, striking disparities emerge in almost every health category for Antelope Valley (SPA 1), a semirural region in Northern LAC.

**Figure 2:**
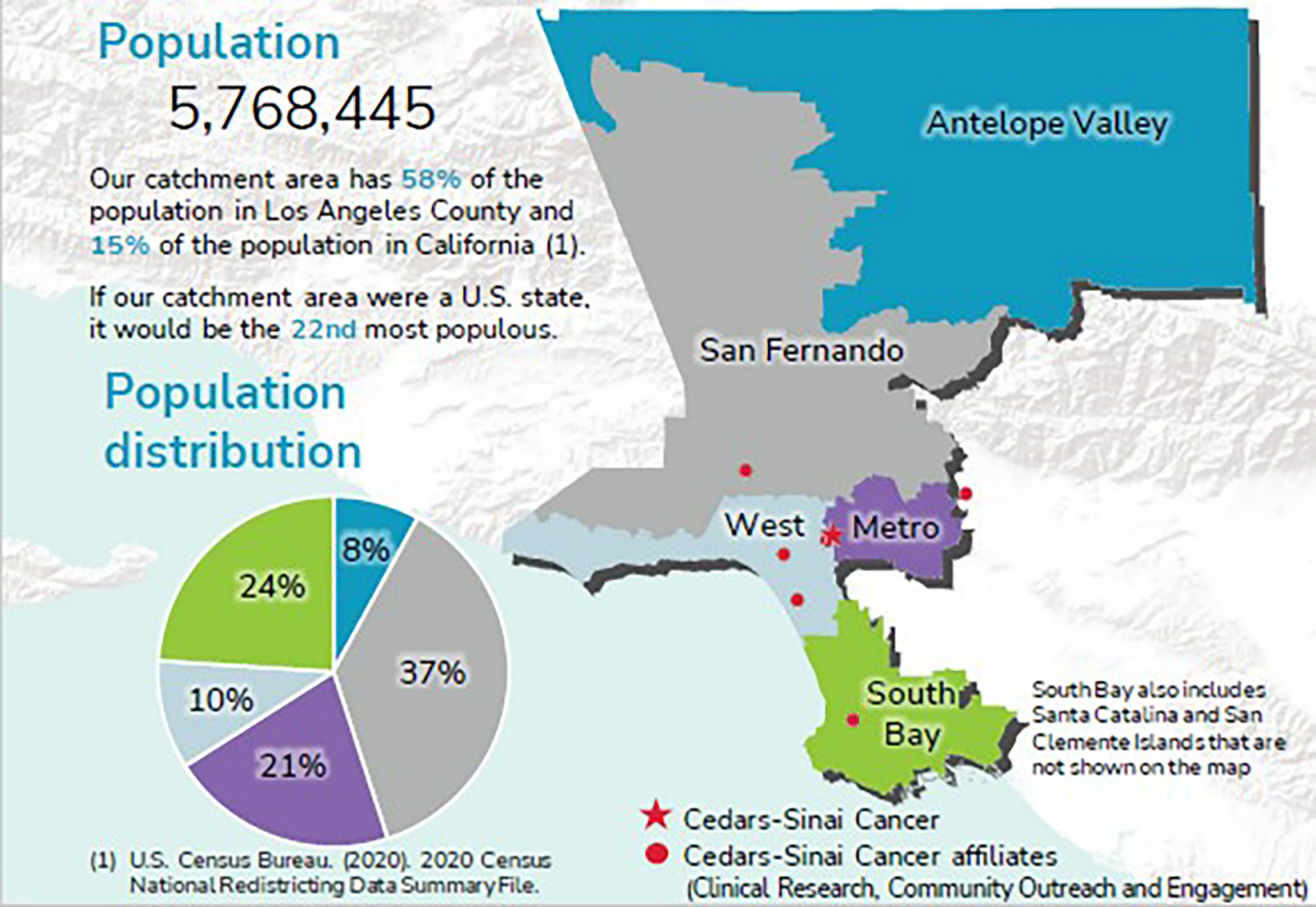
Cedars Sinai Cancer catchment area.

We presented the areas that met the NCI metrics to internal committees at CSC, external advisors, community outreach coordinators, and key decision-makers to ensure that we were aligned with the State’s Cancer Plan and the NCI Catchment Area and Community Outreach and Engagement Mandate. Ultimately, CSC determined its catchment area to encompass service planning areas: Antelope Valley, San Fernando Valley, Metro, West, South Bay (**Figure 2**).

#### Characterizing the Population

**Figure 3:**
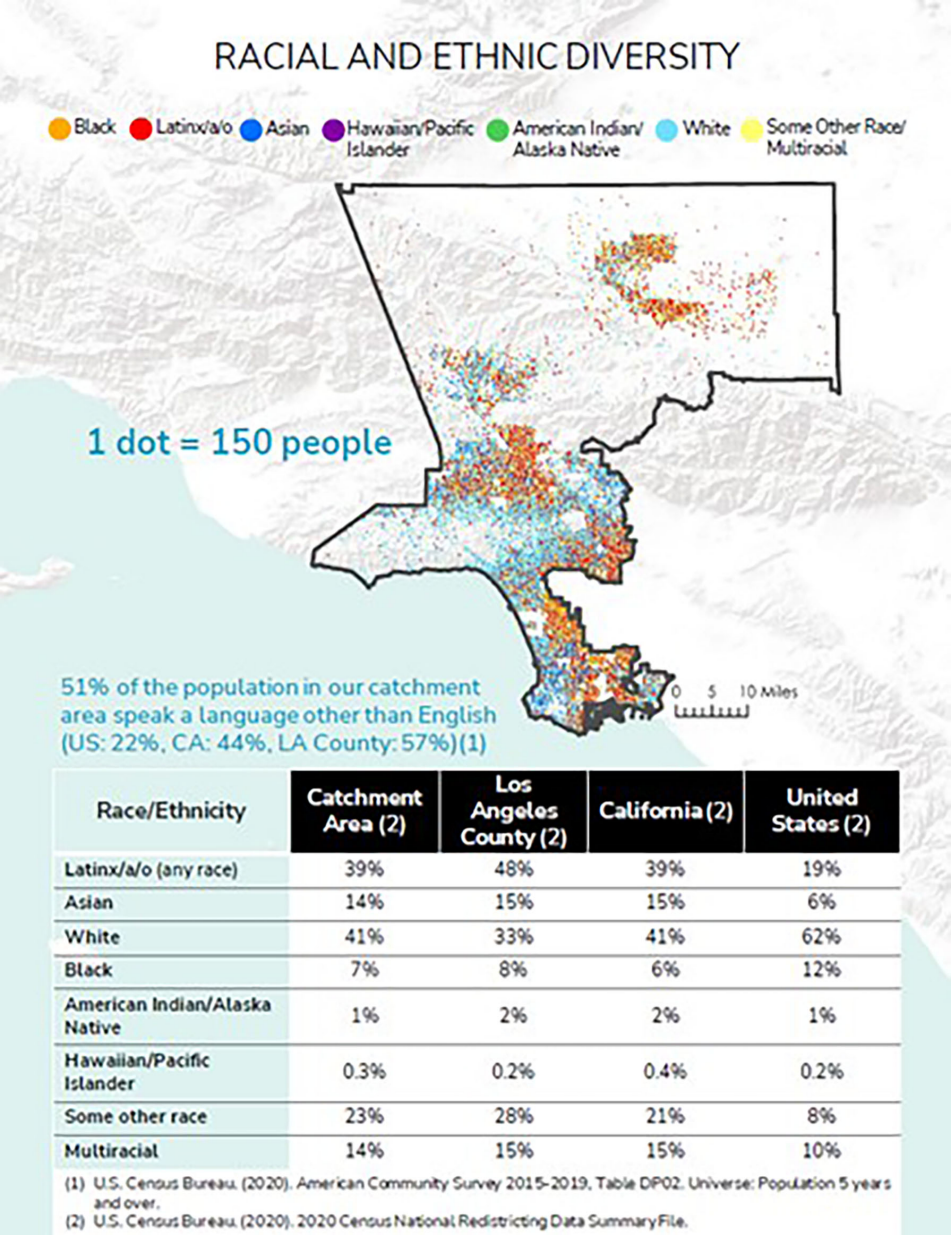
Catchment area population by race and ethnicity.

The selected catchment area has a combined population of 5,768,445 individuals (**Figure 3**).

To gain a better understanding of the catchment area population and its diversity, as well as information on common cancers, secular trends, and mortality, we acquired aggregated and linked data from multiple primary and secondary sources.

##### Secondary Data Collection

First, we started with data at the national level (NCI, Surveillance, Epidemiology, and End Results Program; The Behavioral Risk Factor Surveillance System; American Community Survey; the American Cancer Society) to characterize national trends in cancer incidence and mortality and assess selected behavioral risk factors for populations of interest in our catchment area. Next, we reviewed statewide data from the California Cancer Registry and the California Health Interview Survey (CHIS). CHIS is the nation’s largest state health survey and is conducted by the UCLA Center for Health Policy Research. At the time, the CHIS data cycle did not include comprehensive cancer screening questions; however, we have now partnered with CHIS to include cancer screening questions in the next data cycle (2021-2022) and to oversample CSC’s catchment area populations of Latinx and Asians age 50+ to increase the number of participants in these groups. At the county level, we collated information from the Los Angeles County Cancer Surveillance Program and Los Angeles County Department of Public Health. Finally, we applied innovative geospatial mapping of these data to identify local hotspots for screenable cancers diagnosed at late stages with overlays of other relevant data (e.g., density and location of Federally Qualified Health Centers – FQHCs) to better understand local cancer disparities (discussed below).

##### Primary Data Collection

We supplemented our secondary data collection with additional questionnaires to better understand barriers to adherence that individuals face with cancer preventionand early detection efforts. We focused on social and behavioral risk factors in different racial and ethnic pockets through the administration of culturally adapted questionnaires, as exemplified with the Cancer and Healthcare in Los Angeles Survey (CHILAS). The CHILAS survey was developed with input and feedback from large communities in the catchment area (Korean, Filipinx, and Latinx) to identify major factors that characterize and influence screening behavior, medical history, and health care access. To date, a total of 3,200 surveys have been completed. Of these, 381 surveys have been collected from the Korean community, yielding interesting results. For example, the top barriers for not getting screened were not having health insurance and not feeling sick, suggesting the importance of financial concerns and cultural considerations. Also, we observed that mammography rates among age-eligible women (N=216) were low at 37% (N=80), whereas colonoscopy rates among age-eligible participants (N=284) were higher at 64% (N=182), leading us to question: “What are the unique factors for low mammography screening in Korean women in Los Angeles County (that do not apply to colonoscopy)?”

In the Filipinx community, upon review and feedback from our Filipinx community advisory board, the CHILAS survey was further adapted, and a recruitment strategy was implemented to form a Filipinx Cohort. A total of 1,492 surveys were collected from the Filipinx community in two waves. For screenable cancers, in contrast to Koreans, low adherence to colorectal cancer screening guidelines was identified. In waves 1 and 2, we found that 61% (386 out of 629) of age-eligible men and women had ever had an FOBT and/or colonoscopy, and in wave 2 where the question was updated to ask about most recent screening, only 44% (126 out 287) of age-eligible men and women are up to date with colorectal cancer screening (had FOBT within a year and/or had a colonoscopy within the last 10 years). This finding is consistent with the national trend of Filipinx Americans underutilizing life-saving screening tests for colorectal cancer, resulting in later stage of diagnosis and poorer survival (18). With this information, we began to think about how to best increase screening within this population in our local setting.

##### Community Profile Snapshots

With both primary and secondary data, we developed community profile summaries for several racial/ethnic/gender/sexual orientation minority groups. These profiles highlight noteworthy cancer trends, as well as other social determinates of health such as income, poverty, access to health care, mental health, and literacy. Further, we examined risk behaviors such as substance abuse, physical inactivity, and poor nutrition (**Supplement A**).

### Step 2 & 3: Conduct Community Engaged Research That Addresses the Needs of the Catchment Area

Fifteen different cancer disparities were identified from our initial assessment, which has led to several culturally tailored research initiatives designed to address the needs of the catchment area. Below we provide two examples of studies that span the cancer control continuum, from data collection and interpretation, to designing, implementing, evaluating, and disseminating COE research.

#### Late-Stage Breast Cancer

**Figure 4:**
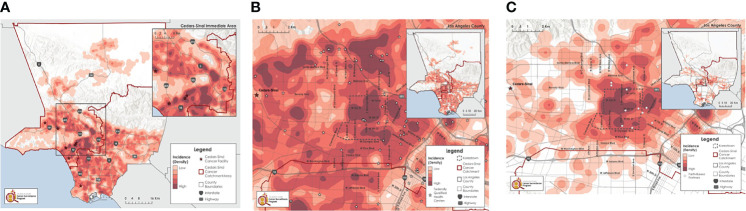
**(A)** All race/ethnicity groups, females, all ages, 2000-2017*. **(B)** Focus on Koreatown, all race/ethnicity groups, females, all ages, 2000-2017*. **(C)** Focus on Koreatown, Korean, females, all ages, 2000-2017*.

In partnership with the Los Angeles County Cancer Surveillance Program, we explored the geographic distribution of late-stage cancer for selected cancers for which there are effective screening protocols. Analysis that examined the geographic distribution of late-stage breast cancer in Los Angeles County found that, using cancer registry data from 2000 through 2017, the densest concentration of late-stage breast cancer for all racial/ethnic groups combined was in our catchment area (**Figure 4A**). In Metro (SPA 4), analysis at finer geographic resolution showed that the Koreatown area has one of the densest concentrations of late-stage breast cancer among all race/ethnic groups (**Figure 4B**). These high-density areas were near many Federally Qualified Health Centers (FQHCs), noted by stars in **Figure 4B**, that offer free or low-cost breast cancer screening services. This includes the Every Woman Counts program funded by the State of California, indicating that these communities remain underserved despite high geographic accessibility to care. Koreatown is one of the few local neighborhoods in Los Angeles County where populations of Korean ancestry predominantly live. To effectively reach these individuals, promote early breast cancer screening, and encourage the use of free or low-cost screening services, our community outreach coordinator conducted in-language workshops in partnership with churches (noted by grey circles in **Figure 4C**). Through these workshops, subsequent focus groups, and existing literature, cultural barriers were identified as an important factor in screening adherence in this population (19). Some of these barriers include: lack of insurance, poor health literacy, not knowing where to go to get screened, lack of follow-up care, fear of being a burden to the family, and inability to afford testing. Another significant challenge was limited English proficiency, which is problematic for navigating an already complicated healthcare system, especially for those who are uninsured or underinsured (20, 21).With this information and building on a network of churches in Los Angeles that have committed to cancer prevention and control activities, grant funding was secured through the California Breast Cancer Research Program to answer the question: Does a culturally adapted “Faith in Action!” curriculum to educate and certify lay health navigators to provide breast cancer screening navigation within faith-based settings increase the adherence to breast cancer screening guidelines among Korean American women? The project is examining an innovative, culturally adapted cancer screening training for lay health navigators to increase adherence to breast cancer screening guidelines among underserved Korean American women. Navigation includes facilitation of follow-up care for those who have an abnormal mammography result, clinical breast exam findings, or are diagnosed with breast cancer. We work closely with members of our Korean Community Advisory Board (CAB), an extensive network of community partners established through our Health and Faith Initiative, to help us articulate the voice of the community to program staff by advising on projects and activities conducted by the research team and providing input to the overall project. Specifically, we have worked together to: (1) refine and finalize the adapted Cancer 101 cancer education training curriculum; (2) participate in decision making for planning of the study development and implementation and help with recruitment based on their knowledge of the population; (3) review the progress of the study; (4) provide guidance on developments in the community that could affect intervention implementation; (5) contribute to interpretation of study findings; and (5) participate in dissemination of study findings. A pastor of one of the larger churches serves as a multi-PI on this intervention study. This is one example of community-engaged research conducted to address the cancer burden in our catchment area that provides the community with a strong voice in all aspects of the study.

#### Melanoma

In the United States (US), melanoma mortality rates have declined by nearly 18% since 2014 in non-Latinx White (NLW) individuals (2); however, similar trends are not apparent in those of lower socioeconomic status (SES), including the Latinx community, and those living in rural areas (3–10). This may be attributed to less access to the information and services that are critical for preventing, detecting, and treating melanoma.

Data from the California Cancer Registry and other literature show that the melanoma burden is increasing in Latinx adults in California, who represent the largest ethnic group in the state, at 39% (11), and typically presents with more advanced disease (8, 13, 14, 22, 23). While US melanoma incidence rates remain low among Latinx adults compared to NLWs (4.6 vs 24.9 per 100,000 from 2012-2016), melanoma mortality is higher compared with other non-white racial/ethnic groups (13, 15). Differences in primary melanoma location (leg/hip/foot) and clinicopathologic subtypes (acral and nodular) in Latinx adults compared with NLWs tend to hamper early detection (8, 15–17). Likewise, physician- and self-skin examination is reported at lower rates in Latinx adults compared to NLW adults (18). In collaboration with Stanford University, we conducted focus groups among low socio-economic and/or Latinx individuals in both urban and rural communities across California to better understand awareness of melanoma prevention and screening practices, and to obtain feedback on primary and secondary prevention strategies in local communities. The interview topics included: 1) awareness and views of melanoma risk, prevention, and early detection screening practices; 2) acceptability of primary and secondary prevention strategies in their respective community; and 3) barriers and facilitators of engagement in melanoma prevention and care. Using a hybrid inductive and deductive approach, thematic analysis was used for data analysis. Findings were organized within a socioecological model (individual, interpersonal, community and health system/policy level). These factors include ethnicity, cultural and gender identity, geography, skin color, gender norms, socioeconomic status, lack of trust, and insufficient access to health care. Latinx participants and those living in semi-rural regions reported more barriers (24). As a result, we are now working with the California Cancer Registry to ascertain individuals in these populations who have been recently diagnosed with melanoma, plus their network of family and friends in both high-density (Bay Area, City of Los Angeles) and semi-rural communities (Salinas, Antelope Valley).This pilot intervention, which includes innovative health communications such as storyboard sketches and whiteboard animations using plain language, as well as use of teledermoscopy through mobile devices, was designed with feedback on early concepts to ensure the communications will reach the target audiences. We are testing the efficacy of a culturally and linguistically appropriate health education intervention, delivered by trusted messengers such as community health workers, to promote melanoma prevention and early detection alongside health care navigation. This is another example of the research conducted within our catchment area that directly responds to the needs of the population, this one introducing innovative design, navigation, and teledermoscopy to address reported barriers.

In summary, we used the following process to identify cancers and behaviors of primary, initial focus. 1) We considered publicly available data such as cancer registry data (SEER, California Cancer Registry, LA County Cancer Surveillance Program), with a focus on top five cancers and cancers with increasing incidence rates, such as liver cancer in Latinx and breast cancer in selected Asian populations. 2) We generated our own quantitative data from: a) conducting geospatial analyses of cancer registry data; b) conducting our own survey, CHILAS, described above; c) sexual and gender minority questionnaire; and d) the California Health Interview Survey (CHIS) described above. 3) We also conducted study-specific surveys when useful. 4) In addition, we continue to seek qualitative input from community advisory boards (CABs, described below), townhall meetings, media events, and participation at community events, such as PRIDE events in greater LAC to identify issues of concern to them and to set priorities. 5) We considered strengths at Cedars-Sinai Medical Center (CSMC) that we may leverage to address specific disparities. 6) We aligned our efforts with the NCI Catchment Area and Community Outreach and Engagement Mandate and the State of California’s 5-Year Cancer Plan.

## Results

**Table 1** lists important cancers/behaviors/disparities, as identified by the community and our quantitative analyses, with consideration of our strengths at CSMC using the process we described above.

**Table 1:** Noteworthy Disparities/Risk Behaviors in Current CSC Populations of Scientific Impact.

Population	Cancer Disparity	Social and Behavioral Risk Factors
Latinx	‣ Liver cancer ‣ Late-stage melanoma ‣ Colorectal cancer	‣ Obesity ‣ Physical inactivity
Korean	‣ Breast cancer ‣ Colorectal cancer ‣ Thyroid cancer	‣ Low screening compliance ‣ High rates smoking/alcohol
Filipinx	‣ Thyroid cancer ‣ Breast cancer ‣ Prostate cancer	‣ Obesity ‣ Low screening compliance ‣ Smoking
Black	‣ Prostate cancer ‣ Triple-negative breast cancer ‣ Pancreatic cancer	‣ Smoking ‣ Secondhand smoke
LGBTQ+^2^	‣ Lung cancer ‣ HPV-related cancers	‣ Medical mistrust/discrimination ‣ HPV awareness & vaccine uptake ‣ Smoking, other drug use ‣ Transgenders: adverse health behaviors ‣ Cancer screening disparities
Non-Latinx Whites	‣ Late-stage melanoma	‣ Low SES cancer screening disparities

### Step 4: Address Disparities

Through partnerships with churches, community organizations, Federally Qualified Health Centers, non-profit organizations, and trained community navigators, our COE team has reached over 16,000 community members of Filipinx (18%), Latinx (20%), LGBTQ+ (25%), Korean (27%), and African American and other (10%) descent in our catchment area with science-based tailored cancer information in our newly defined catchment area. Based on pre/post workshop surveys, there was an 84% improvement in knowledge, behavior, and attitudes concerning cancer risk and prevention for all groups if a community member attended community outreach events, while reduction in barriers to cancer screening was most effective through navigator/promotora training. Knowledge of cancer risk and prevention was also shown to have improved more if there were physical events (33.3%), compared to virtual events (16.1%). With this feedback on our COE strategies, we have a more narrowed focus on a smaller set of cancers and behaviors and have built toward step 4 of NCI’s guidelines, addressing disparities, by developing culturally sensitive, sustainable, scalable, and exportable interventions. We are investing in areas where we believe we have the potential to make a difference in either incidence (long term), mortality, or survivorship experience.

For the Korean breast cancer example, we started by noting the increasing incidence from cancer registry data, increased density of late-stage breast cancer in Koreatown (from our geospatial mapping), and low adherence to breast cancer screening guidelines from our CHILAS data. The grant-funded intervention we describe was facilitated by the CAB and utilizes capacity building among our community partners, training of navigators, and workshops and media events to increase awareness of this issue in the Korean community.

For the melanoma example, we noted that melanoma mortality rates in the US are highest among older men and individuals of lower socioeconomic status. Our findings from our qualitative exploratory study have enriched existing data regarding inequities in lower SES Latinx and non-Latinx White (NLW) individuals and have been critical in designing current interventions that deliver more effective primary and secondary melanoma prevention for underserved populations across geographic regions. At the healthcare systems and health policy level, this work adds to infrastructure and models for collaboration, and is aligned with the Wipe Out Melanoma - California statewide initiative, which is increasing the number of research studies, clinical trials, educational campaigns, and opportunities for the community to engage in melanoma prevention and early detection.

Key to step 4 in the NCI guidelines is the continued involvement of the population in setting a research agenda, and reaching out to the population through research, outreach, and education. The entire research portfolio developed as a result of the methods employed in this paper span across basic, clinical, and population science to guide cancer control and prevention efforts. Through further development of our community advisory boards, and a bidirectional relationship between community outreach and education and research, the populations in the catchment area are at the center of our endeavors. As part of an ongoing assessment process, community leaders representing populations with cancer disparities serve on four active community advisory boards:1) LGBTQ+ Community Advisory Board, 2) Filipinx Community Advisory Network, 3) Latinx Community Advisory Board, and 4) Korean American Community Advisory Board. Representatives from these advisory boards and networks comprise a larger 22 member Cedars-Sinai Cancer Community Advisory Board, which meets quarterly that helps to maintain engagement, guide research into policy implementation and standards of practice, and facilitate translational research across CSMC.

#### Policy and Standard of Practice

An example of how we are guiding research into policy is through our collaboration with

The California Dialogue on Cancer and their Health Equity Taskforce. The CRCHE faculty and staff were instrumental in writing for the first time a section on LGBTQ+ and cancer. Given that there are no reliable cancer registry data stratified by sexual and gender minority status, the CRCHE has been engaged in advocacy efforts to help expand the California Cancer Registry’s data dictionary to include these important variables. The efforts are currently underway with support from the State’s Comprehensive Cancer Control Program which will ultimately impact Surveillance, Epidemiology and End Results (SEER) to become more inclusive of LGBTQ+ populations. This important effort will enable organizations and cancer centers to develop a standardized and coordinated cancer control and research agenda to better serve this population.

#### Community Outreach and Engagement and Translational Science

For COE to inform and facilitate research in the other research programs, Cancer Biology (CB) and Experimental Therapeutics (ET), senior leadership at CRCHE work closely with the CSMC Executive Committee and Leadership Council, which includes other Associate Directors, program co-leaders, and program members where COE and catchment area topics are addressed on a regular basis. These meetings are exclusively focused on COE and the catchment area. An example that emerged from our meetings with ET is a community-based study of Nonalcoholic fatty liver disease (NAFLD) in the Latinx population that is currently a cross-sectional study of NAFLD prevalence that will facilitate a future, planned intervention trial. An example for CB is the initiative to use organoid models to address selected cancer disparities, such as breast cancer in transgender subjects with a focus on hormones and sex differences in bladder cancer.

## Discussion

In this paper, we highlighted how CSC, through guidance from the NCI catchment area framework, has aimed to address health disparities in historically underserved communities. The approach to research and population engagement (steps 2 and 3) has allowed us to work towards solutions that address disparities and aim to alleviate the cancer burden (step 4). Although we have not yet reached the stage of presenting catchment area-level results, our work has led to funded grants that are implementation science based and are presently in the implementation and evaluation phase. The next step currently underway is step 5, which focuses on the representation of the catchment area population in clinical trials. Inclusion of racial and ethnic minorities in cancer clinical trials is critical to increasing the generalizability and knowledge of the risks and benefits of new interventions; however, evidence points to low participation among racial/ethnic minority populations (25–27). in response, CRCHE continues to consult with our CABs, cancer survivorship groups, and coalitions to identify and address barriers for participating in clinical trials, as well as identify opportunities within our existing initiatives to increase accruals, including partnerships with providers, FQHCs, employer groups, and community organizations.

Each cancer center faces its unique challenges in defining, characterizing, and addressing the needs in their catchment area population. We have found that there is not a ‘one size fits all’ approach, especially in regions such as Los Angeles County that have diverse populations with pockets of dense, ethnic enclaves. Approaches must aim to be sensitive and inclusive of all races, ethnicities, and sexual and gender identities, a goal achieved through continuous tailoring and community engagement. A consideration of multiple health domains and socio-ecological influence is required, as well as a continued and localized assessments of cancer needs and disparities coupled with understanding of racial/ethnic-specific and localized cancer-relevant social and behavioral risk factors; otherwise, trends can be missed, and disparities can widen. Our mixed-methods approach to implementing that framework set forward by the NCI, in concert with continued community outreach and education partnerships, provides a narrative for other cancer centers aiming to create a sustained population-level reduction of cancer burden for individuals and communities experiencing health disparities.

## Data Availability Statement

The original contributions presented in the study are included in the article/**Supplementary Material**. Further inquiries can be directed to the corresponding author.

## Author Contributions

LF, CS, RH, ZS contributed to conception and design of the study. LE, MC contributed to data curation and visualization. LF wrote the first draft of the manuscript. All authors contributed to manuscript revision, read, and approved the submitted version.

## Funding

The late-stage breast cancer study is funded by California Breast Cancer Research Foundation (CBCRP) Award Number B27BB4290; the melanoma study is funded by Mary E. Brenneisen Fund at Stanford Medicine and in part, the National Center for Advancing Translational Sciences of the National Institutes of Health, Award Number UL1TR003142. Additionally, this manuscript is the result of work supported by resources at Cedars Sinai Cancer in Los Angeles, California.

## Conflict of Interest

The authors declare that the research was conducted in the absence of any commercial or financial relationships that could be construed as a potential conflict of interest.

## Publisher’s Note

All claims expressed in this article are solely those of the authors and do not necessarily represent those of their affiliated organizations, or those of the publisher, the editors and the reviewers. Any product that may be evaluated in this article, or claim that may be made by its manufacturer, is not guaranteed or endorsed by the publisher.

## Footnotes

1 Latinx is a term used to represent Latino/a/x populations.

2 We use the term lesbian, gay, bisexual, transgender, queer or questioning, plus anyone else that considers themselves part of this community (LGBTQ+) to encompass the diversity of this population.

## Abbreviations

CRHCE, Cancer Research Center for Health Equity; CSMC, Cedars Sinai Medical Center; COE, community outreach and engagement; LAC, Los Angeles County.
