# Supplementary material for: Addressing Health Disparities Across the Cancer Continuum—a Los Angeles Approach to Achieving Equity
Source: Front Oncol. 2022 Jul 5;12:912832. doi: 10.3389/fonc.2022.912832 (PMC9295745; doi:10.3389/fonc.2022.912832)
Supplement: Supplementary file 1 [file DataSheet_1.pdf]

Supplement A

Cancer Research Center for Health Equity Community Profiles: LGBTQ+, Latinx, Korean, Filipino, Semi-rural (Antelope Valley)

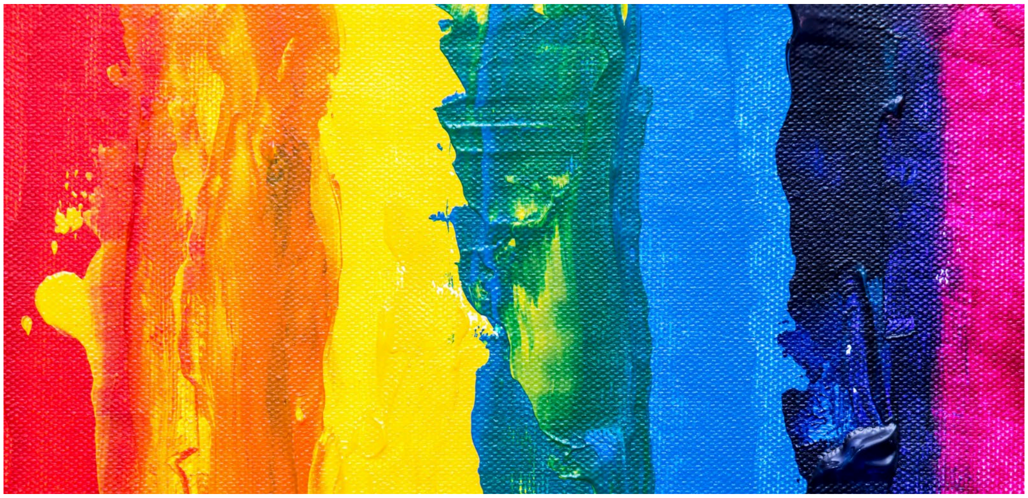

Community Profile: LGBTQ in Los Angeles County

PUBLISHED BY THE CANCER RESEARCH CENTER FOR HEALTH EQUITY AT CEDARS-SINAI

Photo by Steve Johnson on Unsplash

Community Overview

Population

10 million American adults identify as lesbian, gay, bisexual, or transgender (LGBT)* (4.1%). Los Angeles is second only to New York in the total number of individuals who identify as LGBT at 590,000[1].

Age and Gender

Millennials are more than twice as likely as any other generation to identify as LGBT. In 2012, they accounted for 43% of the LGBT population (see Figure 1). In 2012, 3.5% of women identified as LGBT and by 2016, that number increased to 4.4%, compared with 3.7% among men[1].

Race and Ethnicity

Since 2012, Asians and Hispanics have experienced the largest increase in LGBT identification compared to other racial and ethnic minorities, 3.5% to 4.9% among Asians and 4.3% to 5.4% among Hispanics[1]. Among whites, the comparable figures are 3.2% to 3.6%. Black Americans showed only a slight increase from 4.4% to 4.6%, and among "other" racial and ethnic groups, the increase was from 6.0% to 6.3%[1].

* We use the term LGBT to encompass the diversity of this population. However, gender and sexual minority status have not been regularly collected in national surveys, therefore acronyms throughout this application reflect that specific subset of the population for whom data were available (i.e., LGBT, LGB, T, LGBTQ+).

FAST FACTS

Over the past five years, Americans who identify as LGBT are younger, more female, and less religious than their counterparts[1]

Income

LGBT Americans are more likely to be poor compared to non-LGBT individuals[3]. Further, race/ethnicity, gender, geography, and education influence poverty rates among LGBT populations; children of same-sex couples are particularly vulnerable to poverty. In California, LGBT females tend to do worse than their male counterparts on socioeconomic indicators such as income and whther they have enough money for healthcare[4].

Poverty

Compared to the general population, LGBT people are disproportionately poor overall, although there is variation between subgroups. A 2013 Pew Research poll of LGBT individuals found that about 4 in 10 (39%) earned $30,000 or less per year, compared to 28% of the U.S. population overall. Poverty rates on average are higher among lesbian and bisexual women, young people, and African Americans[5].

Transgender and Access to Care

A 2015 survey of 27,715 transgender people from across the U.S. found that one-third (32%) of respondents had an annual income of less than $10,000 compared to 23% of the US population.[4, 6]. Approximately 30% of LGBT adults do not seek healthcare services or lack a regular healthcare provider compared with 10% of age-matched heterosexuals,[5, 7, 8] and 23% of transgender respondents did not see a doctor when they needed to because of fear of being mistreated as a transgender person.[9]

Insurance

During the early years of ACA implementation, the rate of uninsurance among LGB individuals fell by almost half (from 19% in 2013 to 10% in 2016), representing an estimated 369,000 fewer uninsured LGB individuals in 2016 compared to 2013. LGB individuals also saw significant gains in Medicaid coverage between 2013 and 2016 (increasing from 7% to 15%). This increase represents an estimated 511,000 more LGB individuals with Medicaid coverage in 2016 compared to 2013[10].

Figure 1

Percentage of U.S. Adults Identifying as LGBT by Birth Cohort, 2012-2016

	2012	2013	2014	2015	2016
	%	%	%	%	%
Millennials (1980-1998)	5.8	6.0	6.3	6.7	7.3
Generation X (1965-1979)	3.2	3.3	3.4	3.3	3.2
Baby boomers (1946-1964)	2.7	2.7	2.7	2.6	2.4
Traditionalists (1913-1945)	1.8	1.8	1.9	1.5	1.4

GALLUP DAILY TRACKING

Reprinted from "In U.S., More Adults Identifying as LGBT" at Gallup https://news.gallup.com/poll/201731/lgbt-identification-rises.aspx

Los Angeles and the LGBTQ Community

At the Cancer Research Center for Health Equity (CRCHE) at Cedars Sinai Cancer (CSC), we are situated at the hub of the LGBT community in Los Angeles County. The West Hollywood area surrounding CSC has the highest self-identified LGBT population in the County at close to 7%, and the area adjacent to CSC has the second highest concentration of LGBT adult population at 6.1%[2], compared to about 4% for the nationally reported LGBT population (see Figure 2).[1] CRCHE has developed an LGBTQ Community Advisory Board (CAB) comprised of 12 community organizations, health care providers, and advocacy groups that represent the diversity within the LGBTQ communities. Over the past two years, CRCHE has been actively engaged with the CAB to understand their challenges, particularly among underserved, low- income, and diverse LGBTQ communities of color, including of African American, Latinx, Asian, Pacific Islander, Armenian, Persian, and Arab descent.

Figure 2

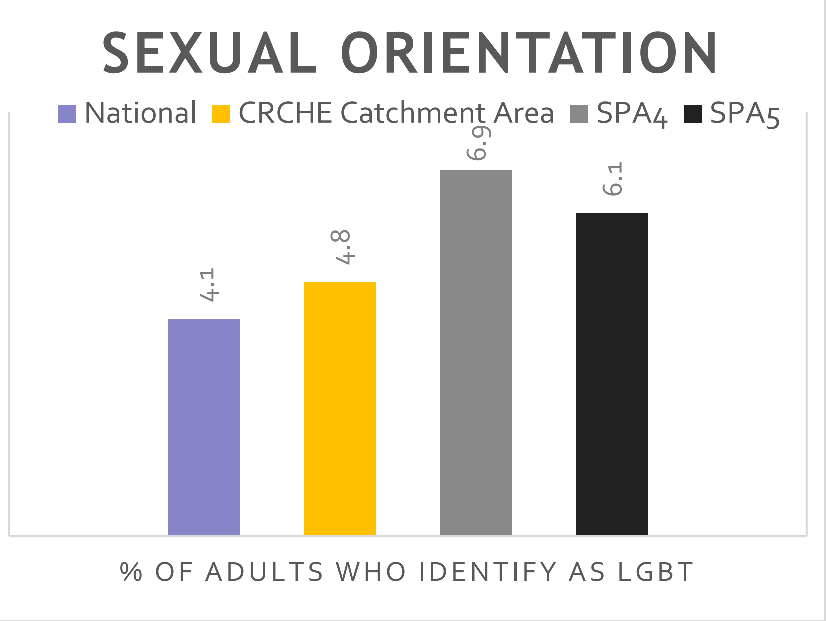

Source: Los Angeles Department of Public Health, Key Indicators of Health by Service Planning Area. January 2017

Site Specific Cancers

Sexual minority status is not regularly collected in national surveys and registries and therefore there may be limited generalizability of these results.

ANAL CANCER

Anal cancer is rare, representing about .4% of all new cancer cases in the United States, and only about 0.2% of men and women will be diagnosed with anal cancer at some point during their lifetime[7]. In 2015, approximately 2640 new cases among men and 4630 new cases among women were anticipated in the US[11]. The annual incidence rates for men and women are 1.5 and 2.0 per 100,000 persons per year, respectively, and the mortality rates for men and women are 0.2 and 0.3 per 100,000 persons per year, respectively[7]. Incidence of anal cancer among HIV-positive MSM (45.9 per 100,000 person-years) is 9-fold higher than among HIV-negative MSM (5.1 per 100,000 person-years), with the latter still higher than that observed in the general population[12].

In one study, HIV-positive men had a significantly higher prevalence of anal cancer (45.9 per 100,000 person-years) compared with HIV-negative men (5.1 per 100,000 person-years) and compared with the overall incidence in the general population (1.5 per 100,000 person-years)[7].

COLON AND RECTAL CANCER

Colorectal cancer is the third most common cancer among men and women in the US, with annual incidence rates of 48.9 and 37.1 per 100,000 persons, respectively. Mortality rate is 18.6 and 13.1 per 100,000 for men and women, respectively[7].
Weighted prevalence estimates found there was no significant difference between heterosexual women (2.8%) versus lesbian women (4.5%) or between heterosexual men (4.4%) and gay (2.7%) or bisexual (1.7%) men. A separate county-level ecological study found that counties with higher density of LGBT tend to have higher incidence of colorectal cancer for both men and women, although there is question as to reliability and generalizability of this data[7].

LUNG CANCER

Lung cancer is the second most common cancer and the leading cause of death in the US. Annual incidence is 70.1 per 100,000 for men and 50.2 per 100,000 for women; annual mortality rate is 59.8 per 100,000 and 37.8 per 100,000 for men and women, respectively[7]. County-level ecological studies that have been conducted found modest differences in lung cancer risk in the LGBT community, but it is not possible to draw conclusions linking sexual minority status and cancer risk at the individual level. Until gender orientation and sexual minority status are collected at the local, state, and federal levels, incidence levels of lung cancer will remain theoretical or estimations. We do know that smoking in the LGBT community is higher compared to heterosexuals, with rising rates among LGBT youth[7].

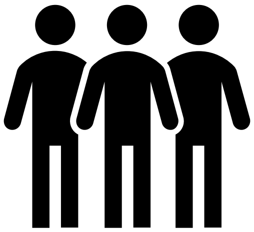

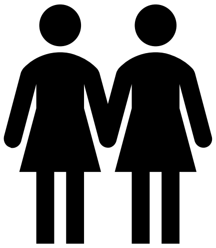

FAST FACTS

Throat Cancer

Within 20 years, health experts expect the majority of head and neck cancers to be caused by HPV-positive carcinomas instead of smoking and alcohol, and by 2020, the rates of HPV-related oropharyngeal (area encompassing the throat, tonsils, and back of the tongue) cancer will surpass those of cervical cancer[13]

BREAST CANCER

Breast cancer is the most frequently diagnosed cancer in women. The estimated annual incidence of female breast cancer is 124.8 per 100,000 women per year and mortality rate is 21.9 cases per 100,000[7]. In 2015, American Cancer Society estimated that there would be approximately 2350 new cases of invasive male breast cancer and about 440 men would die from breast cancer. There is no published data on breast cancer incidence in the LGBT community, and the few published prevalence estimate studies are small and poorly designed. Researchers have used novel approaches to try and estimate prevalence, incidence, and mortality of breast cancer in the LGBT population and found that prevalence of breast cancer did not significantly differ by sexual orientation[7].

CERVICAL CANCER

The incidence of cervical cancer in the US is approximately 7.7 cases per 100,000 women, and mortality is 2.3 per 100,000. To there are no published data on cervical cancer incidence and mortality among lesbian and bisexual women. However, weighted prevalence estimates show that heterosexual women have a significantly lower prevalence of cervical cancer (14%) compared with lesbian women (16.5%) and bisexual women (41.2%)[7].

HPV infection is present in nearly all cervical cancer cases and is the most important risk factor for cervical cancer.

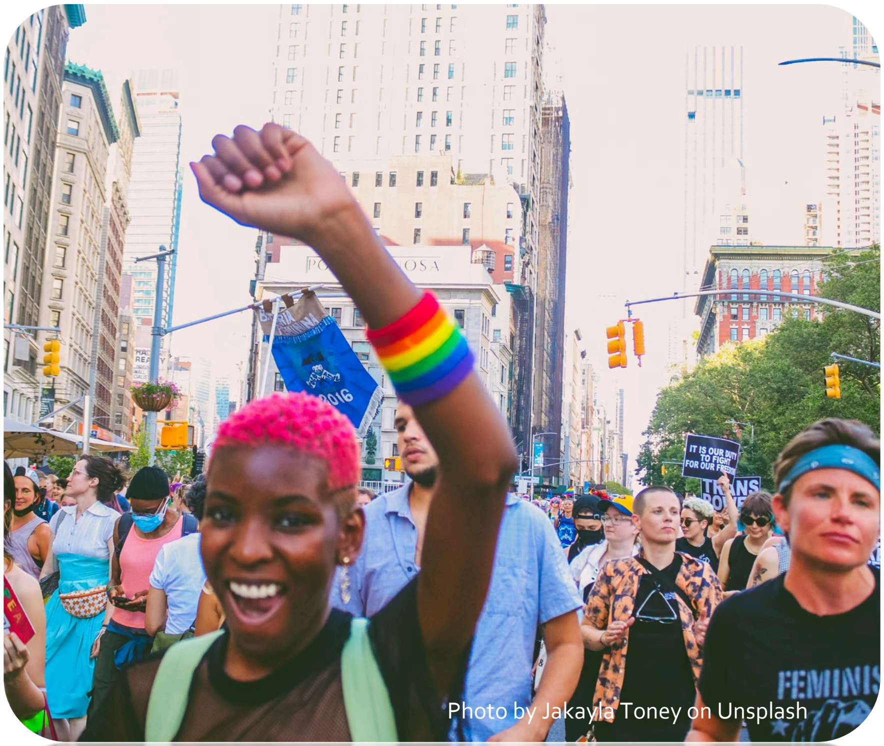

Photo by Jakayla Toney on Unsplash

Risk Behaviors

Risk behaviors that potentially contribute to elevated cancer risks for the LGBT population include differences in parity, smoking status, cancer screening, and access to care. Rates of smoking are higher in LGBT communities compared to other populations, which may increase the risk for 12 cancers caused by tobacco use. Disparities in screening behaviors in LGBT communities have also been identified and need to be more fully addressed in future efforts[8, 14].

Infectious Diseases

HPV is a casual risk factor in the development of anal cancer and is present in greater than 80% of cases of anal squamous cell carcinoma. Other risk factors include a high number of lifetime sexual partners, MSM, multiple and concurrent sexual partners, coexistence of other sexually transmitted infections, cigarette smoking, and immunosuppression[7]. Because MSM are at a higher risk for HPV-associated anal cancer compared to heterosexual men and women, they are an important target group for HPV vaccination [7, 14].

HIV status has been linked to higher cancer prevalence and mortality rates in some cancers. In one study, cancer and HIV registries were linked in six states and showed higher cancer mortality in HIV-positive patients compared with HIV-negative patients for colorectal, pancreas, larynx, lung, melanoma, breast, and prostate cancers. HIV-positive patients continued to have higher cancer mortality rates in colorectal, melanoma, and breast cancers, even after adjusting for cancer treatment [7, 14].

Tobacco, Alcohol, and Recreational Drug Use

LGBT populations report higher tobacco, alcohol, and recreational drug use than the heterosexual population. Bisexual men and women report the highest rates of alcohol use and alcohol-related problems compared with heterosexual counterparts. Cigarette smoking remains the most preventable cause of cancer death. Cigarette smoking increases risk of cancers of the oropharynx, larynx, lung, esophagus, pancreas, uterine cervix, kidney, bladder, stomach, colorectum, and liver, as well as acute myeloid leukemia. There is also a causal association of alcohol consumption with cancers of the oropharynx, larynx, esophagus, liver, colon, rectum, and female breast. Clinicians should encourage any and all interventions for patients to quit tobacco use (e.g., pharmacologic, nicotine replacement [patch, gum, lozenge], hypnosis, acupuncture, self-help books, quit lines, etc.)[7, 14].

For substance abuse, it is estimated that 20% to 30% of gay and transgender people abuse substances, compared with about 9% of the general population. This may be a way to cope with the stresses of discrimination and stigma. Clinicians should offer support and appropriate referrals to patients who use substances. Substance use may be tied to underlying depression or other psychological concern and so, as noted previously, it is key to address psychological problems that may occur as well[7, 14].

Access to Care and Cancer Screening

Cancer screening recommendations by the US Preventive Services Task Force (USPSTF) have historically been gendered, causing confusion for non-binary and transgender individuals.[15-17] Cancer screening for breast, cervical, colorectal, prostate, and lung cancers are effective tools to reduce morbidity and mortality of these cancers; screening for cervical and colorectal cancers may prevent cancers by finding precancerous lesions and polyps, respectively. The USPSTF recommends screening for cervical (women 21-60 years; Grade A), colorectal (men and women 50-75 years; Grade A), breast (women 50-74 years; Grade B), and lung (men and women 55-80 years with smoking history; Grade B) cancers.[18] For prostate cancer, the recommendation is for men 55-69 (Grade C) to make a decision regarding screening after discussion with their clinician, balancing individual potential hazards and benefits on the basis of family history, race/ethnicity, co-morbid conditions, etc.[19] **LGBTQ communities have lower rates of participation in cancer prevention interventions (see Table 1), resulting in missed opportunities for primary and secondary cancer prevention that lead to late detection of cancer, and reduced post-treatment follow-up care.**

Table 1. LGBT groups with lower prevalence of cancer screening compared to cisgender or heterosexual groups

Authors (data source)	Breast	Cervical	Colorectal	Lung	Prostate
Tabaac et al, 2018 (BRFSS)	Transgender women; GNC	Transgender men; GNC	Transgender women; GNC	--	Transgender women; GNC
Veliz et al, 2019 (BRFSS)				Lesbian, Bisexual women	
Charkhchi et al, 2019 (BRFSS)	Bisexual, Male-to-female transgender, GNC	Lesbian/gay, female to male transgender, GNC	Male to female transgender	--	--

GNC=gender non-conforming

Highest Risk: People of Color, Transgender, Low SES, Overall Well-Being

When evaluating the LGBT community, attention must be paid to the highest risk groups within this minority population. Transgender individuals are perhaps the most marginalized minority group in the LGBT community. Transgender patients frequently report experiencing discrimination in health care settings, and face a health system that lacks personnel who are adequately trained and competent in transgender medicine.24 The National Center for Transgender Equality national survey found that 39% of transgender people reported having experienced serious psychological distress.23 In addition, 40% of transgender people reported having attempted suicide in their lifetime, and 7% attempted suicide in the past year.

About the Cancer Research Center for Health Equity

In 2018, Cedars-Sinai and Samuel Oschin Comprehensive Cancer Institute expanded their research enterprise to include a new center focused on addressing cancer disparities in the community through research, service, and policy. The Cancer Research Center for Health Equity aims to conduct research that is well integrated with community engagement and outreach efforts to reduce cancer incidence/mortality in underserved populations and neighborhoods in Los Angeles County.

References

1. Gallup. *In U.S., More Adults Identifying as LGBT*. Social and Policy Issues 2017 May 2018; Available from: http://news.gallup.com/poll/201731/lgbt-identification-rises.aspx?g_source=Social%20Issues&g_medium=newsfeed&g_campaign=tiles.

2. Los Angeles County Department of Public Health, *Key Indicators of Health by Service Planning Area*, O.o.H.A.a. Epidemiology, Editor. 2017.

3. M.V. Lee Badgett, L.D., Alyssa Schneebaum,,, *New Patterns of Poverty in the LGB Community*. 2013, Williams Institute: Los Angeles.

4. The Williams Institute, *The LGBT Divide in California: A look at the socioeconomic well-being of LGBT people in California*. 2016, UCLA.

5. Kates, J., et al.,,, *Health and Access to Care and Coverage for Lesbian, Gay, Bisexual, and Transgender Individuals in the U.S.* 2018, Henry J Kaiser Family Foundation.

6. The Williams Institute, *Demographics and Health of California's Transgender Adults: FIndings from 2015-2016 California Health Interview Survey*, in *Health Policy Brief*. 2017, UCLA.

7. Quinn, G.P., et al., *Cancer and lesbian, gay, bisexual, transgender/transsexual, and queer/questioning (LGBTQ) populations.* CA Cancer J Clin, 2015. **65**(5): p. 384-400.

8. Burkhalter, J., *The National LGBT Cancer Action Plan: A White Paper of the 2014 National Summit on Cancer in the LGBT Communitities.* LGBT Health, 2016. **3**(1).

9. James, S.E., Herman, J. L., Rankin, S., Keisling, M., Mottet, L., & Anafi, M., *The Report of the 2015 U.S. Transgender Survey*, in *National Center for Transgender Equality*. 2016, National Center for Transgender Equality: Washington D.C.

10. Dawson, L., Kates, J., Damico, A, *The Affordable Care Act and Insurance Coverage Changes by Sexual Orientation*, in *Disparities Policy*, H.J.K. Foundation, Editor. 2018

11. Siegel, R.L., K.D. Miller, and A. Jemal, *Cancer statistics, 2015.* CA Cancer J Clin, 2015. **65**(1): p. 5-29.

12. Machalek, D.A., et al., *Anal human papillomavirus infection and associated neoplastic lesions in men who have sex with men: a systematic review and meta-analysis.* Lancet Oncol, 2012. **13**(5): p. 487-500.

13. McQuillan G, K.-M.D., Markowitz LE, Unger ER., Paulose-Ram R., *Prevalence of HPV in adults aged 18–69: United States, 2011–2014*, in *NCHS data brief*. 2017, National Center for Health Statistics: Hyattsville, MD.

14. Ceres, M., et al., *Cancer Screening Considerations and Cancer Screening Uptake for Lesbian, Gay, Bisexual, and Transgender Persons.* Semin Oncol Nurs, 2018. **34**(1): p. 37-51.

15. Nelson, B., *A cancer screening crisis for transgender patients: Discrimination, patient unease, provider ignorance, and a highly gendered health care system are impeding cancer screening and risk assessment in the transgender population. In this article, the first of a 2-part series, we explore how clinicians can begin to address those barriers.* Cancer Cytopathol, 2019. **127**(7): p. 421-422.

16. Kiran, T., et al., *Cancer screening rates among transgender adults: Cross-sectional analysis of primary care data.* Can Fam Physician, 2019. **65**(1): p. e30-e37.

17. Agenor, M., et al., *Perceptions of cervical cancer risk and screening among transmasculine individuals: patient and provider perspectives.* Cult Health Sex, 2016. **18**(10): p. 1192-206.

18. Force, U.S.P.S.T. *A and B Recommendations*. Recommendation Topics [cited 2020 09/05/2020]; Available from: https://www.uspreventiveservicestaskforce.org/uspstf/recommendation-topics/uspstf-and-b-recommendations.

19. Force, U.S.P.S.T. *Recommendation: Prostate Cancer: Screening*. Recommendation Topics [cited 2020 09/13/2020]; Available from: https://www.uspreventiveservicestaskforce.org/uspstf/recommendation/prostate-cancer-screening.

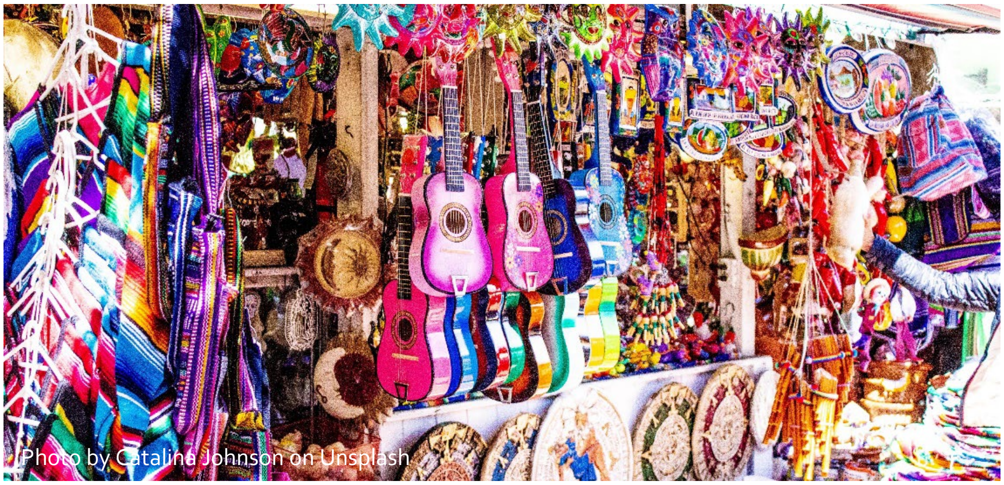

Photo by Catalina Johnson on Unsplash

Community Profile: Latinx in Los Angeles

PUBLISHED BY THE CANCER RESEARCH CENTER FOR HEALTH EQUITY AT CEDARS-SINAI

Community Overview

Population

There are currently about 4,861,648 Hispanic/Latinx/o/a population (hereafter mainly referred to as Latinx) living in Los Angeles County, accounting for 48.4% of the County's total population of 10 million [2]. Among the nearly 4.9 million self-reported Latinx in the County, 76.6% identified as Mexican, 8.7% Salvadoran, 5.2% Guatemalan, 1.1% Honduran, 0.9% Puerto Rican, 0.9% Nicaraguan, 0.8% Cuban, and 2.5% South Americans[2]. 40% of the Latinx living in Los Angeles County are foreign-born [3].

Income

The per capita income among Latinx of $16,940 is much lower than the Los Angeles County average of $28,340. Median household income is nearly $13,000 lower among Latinx compared to the County average, $46,850 and $59,135 respectively. This is 5.7% less than the median Latinx household income across the state and 4.6% lower than across the nation[4].

Sex and Age

Of the 4,861,648 Latinx living in Los Angeles County, roughly 2,437, 468 are female, with 83% being under the age of 55. Of the 2,424,180 Latinx men, 87% are under the age of 55[3].

Education

59.4% of Latinx aged 25 years and older have at least a high school diploma, compared to 77.3% of all other residents in Los Angeles County[4].

Poverty and Unemployment

181, 485 Latinx families are in poverty[1], accounting for 18.5% of all Latinx families in the County[4]. The unemployment rate of Latinx in Los Angeles County is half of a percentage point higher than all other workers in Los Angeles, 7.4% and 6.9%, respectively[4].

Health Insurance Coverage

17.3% of Latinx in the County are uninsured, while 41.7% have public insurance and 41% have private insurance. The overall rate of uninsured in the County is between 9-11%[3, 5].

Mobile Technology

Overall, 80% of Latinx adults say they 'at least occasionally' access the internet via a mobile device such as a cellphone or tablet. While nearly all 18- to 29-year-old Latinx (94%) and 30- to 49-year-old Latinx (89%) use the internet on a mobile device, 58% of Latinx ages 50 to 64 and less than half of those ages 65 and older (35%) do so[6].

Religion

The Pew Research Center's 2013 National Survey of Latinx and Religion finds that a majority (55%) of the US Latinx population identify as Catholic. About 22% are Protestant (including 16% who describe themselves as born-again or evangelical) and 18% are religiously unaffiliated[7].

FAST FACTS

48.4%

Latinx represent the largest ethnic/racial group in Los Angeles[8].

[1] Based on U.S. Census 2015 Federal Poverty Level (FPL) thresholds which for a family of four (2 adults, 2 dependents) correspond to annual incomes of $24,036 (100% FPL). https://www.census.gov/quickfacts/fact/table/losangelescountycalifornia/IPE120216#viewtop

Figures 1 & 2

Most Common Cancers among Latinx in the US

Breast

Breast cancer is the most diagnosed cancer among Latina women. From 2003 to 2012, breast cancer incidence rates stabilized in Latina women. However, breast cancer is still the leading cause of cancer death among Latina women[1].

Colorectal

Colorectal cancer is the second-most diagnosed cancer in both Latinx men and women, with an estimated 6,400 men and 5,300 women in the US diagnosed with cancer of the colon or rectum in 2015. Between 2003 and 2012, death rates for colorectal cancer decreased by about 1.7% per year among Latinx [1].

Prostate

Prostate cancer is the most common cancer among Latinx men, with about 13,000 new cases in the US in 2015. The incidence rate among Hispanics (112.1 per 100,000) is about 9% lower than among non-Hispanic whites (123.0) likely due to lower rates of prostate-specific antigen (PSA) testing among Latinx. From 2003-2012, Prostate cancer incidence rates decreased 4.7% per year in Latinx men[1].

Other cancers

Latinx in Los Angeles County are showing a steadily increasing risk for Hodgkin lymphoma and cancers of the kidney, liver, testis and thyroid[2]. See Figures 1 & 2.

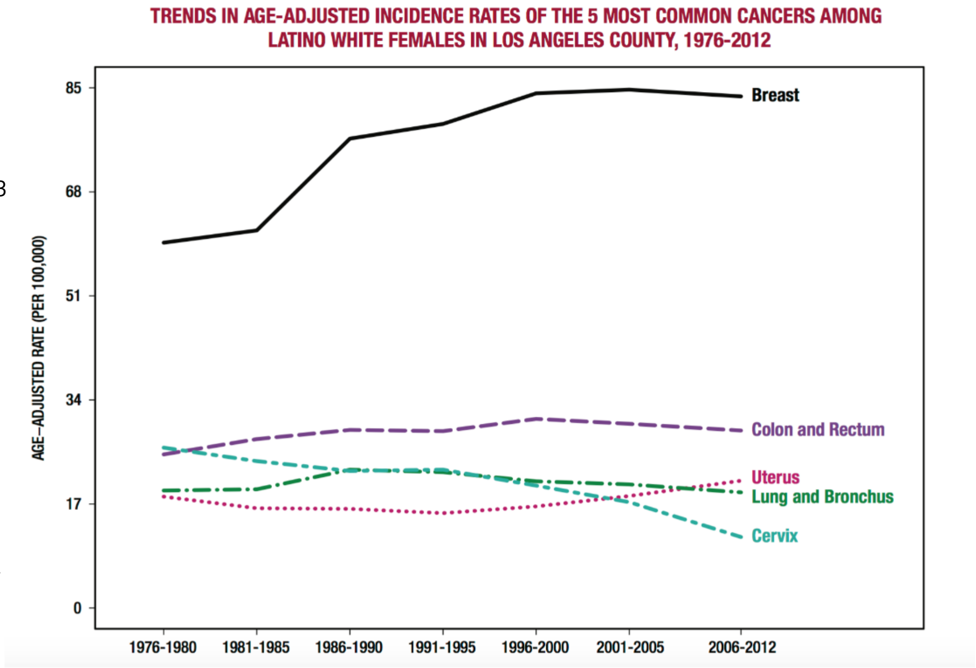

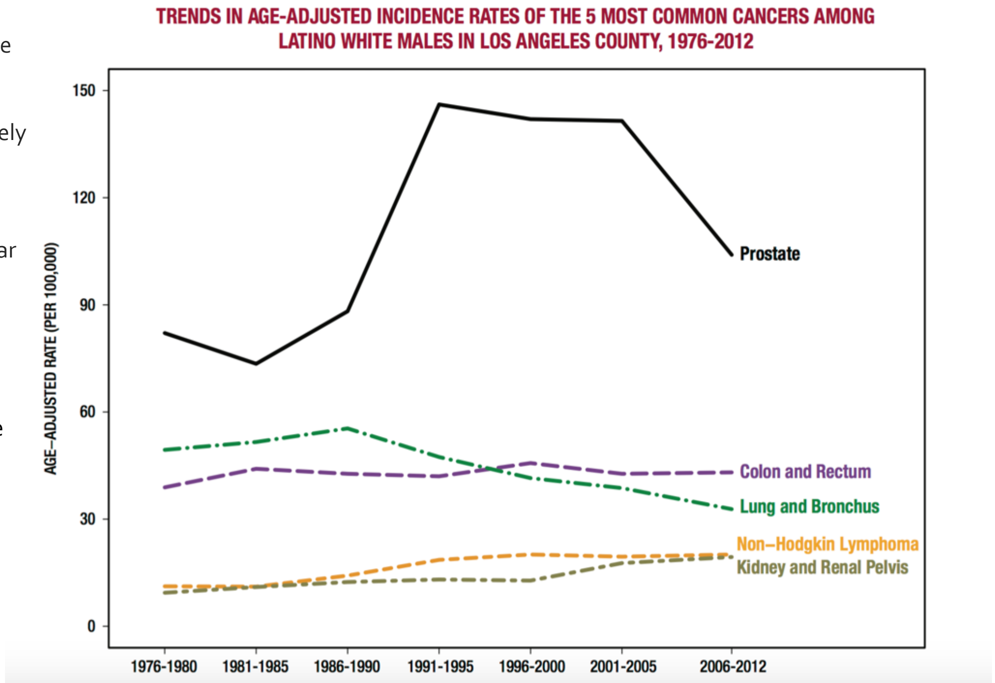

Note. Trends in Incidence Rates Among Latino Males and Females. Reprinted from "Cancer in Los Angeles County: Trends by Race/Ethnicity, 1976-2012," by Liu L, W.Y., Sherman RL, Cockburn M, Deapen D. in *Los Angeles Cancer Surveillance Program*. 2016, University of Southern California. Reprinted with permission.

Trends in Cancer Type in Latinx

As the top five cancers in Latinx men and women are stabilizing, several other incidence trends are on the rise in the Los Angeles Latinx community. Below are standout trends from 1976 to 2012 by cancer type.

LIVER CANCER

Liver cancer death rates are increasing at a faster pace than any other cancer, and liver cancer among Latinx men is forecasted to increase faster than any other ethnic/racial group[9, 10]. Hepatitis B and Hepatitis C are the strongest risk factors for liver cancer worldwide, but obesity and related metabolic disorders, including diabetes, remain the most important risk factors in the United States, as 36.6% of liver cancer is attributed to obesity and diabetes. Most liver cancers are potentially preventable, and interventions to address the growing incidence and racial/ethnic disparities should focus on prevention and early detection, including weight management, access to comprehensive diabetes care, alcohol consumption, tobacco control, and improvements in Hep B and C vaccination, screening, and treatment[10].

OBESITY

And related metabolic disorders like diabetes, remain the most important risk factors in liver cancer.

KIDNEY CANCER

Rates of kidney cancers have increased among Latinx whites of both sexes in the last 40 years. Trends in kidney cancer incidence are limited by the small numbers of cases for some groups, but this pattern follows that of other racial/ethnic groups with increases for all groups in the last decade. One risk factor that might be driving these higher rates is increasing adult obesity rates in the County. However, incidence rates of late stage kidney cancers in Los Angeles, like the rest of the U.S., are fairly stable suggesting this observed increase is at least partially attributable to improved diagnosis[2].

HODGKIN LYMPHOMA

An increasing incidence of all Hodgkin lymphoma was observed among Latina women between 1976-2012, with a minor decrease in the last period of 2006-2012. At a closer look of subtype, nodular sclerosis incidence rates have more than doubled among Latina women. There has been a gradual increase in incidence rates of the nodular sclerosis subtype (associated with young adult disease) in all men, but especially in Latinx men[2]. Epstein-Barr virus (EBV) is seen in tumor cells in about 40% of the cases in the subtype, especially when diagnosed in early childhood and older ages. Populations that are transitioning to higher socioeconomic status are experiencing an increase in this young adult subtype and up to 8% of the risk is explained by genes associated with immune function, including those from the HLA gene family, [2].

TESTIS CANCER

Testis cancer rates among Latinx have risen sharply in recent years. Together with the growing Latinx population in Los Angeles County, these increased rates led for the first time in 2006-2012 to more testis cancer diagnoses among Latinx than among non- Latinx. Environmental influences, habits, and acculturation need to be better studied to provide insights on testis cancer risk. Identifying these causes to mitigate and prevent testis cancer should be a continued focus of testis cancer research[2].

THROID CANCER

In the past few decades, thyroid cancer incidence has been increasing worldwide, with a growing incidence of papillary thyroid cancers. There has been a general increase in the Latinx community with significant increases in the last decade, particularly among adolescents and young adult females ages 15-39[11]. Unique epidemiologic patterns by cell type, sex, and age suggest the increasing trends may be due to an actual increase in etiologic risk, and like kidney cancer, this observed increase is at least partially attributable to improved diagnosis. Analyses by cell type indicates the increase in incidence is observed for papillary, particularly for women, but not other types of thyroid cancers[2].

FAST FACTS

Liver Cancer is highly fatal, and death rates for Latinx in the United States are increasing faster than for any other cancer. Latinx are forecasted to have the highest incidence rates among men and second highest among women by 2030 [9, 10].

Cedars Sinai

Risk Behaviors

For Cancer

Prevention and early detection is key to reducing the cancer burden in the Latinx community, and organized efforts to reduce tobacco use and obesity, improve diet, and increase physical activity and use of established screening tests can save lives [12]. It is estimated that 20% of all cancers diagnosed in the US are caused by a combination of excess body weight, physical inactivity, excess alcohol consumption, and poor nutrition[1, 13].

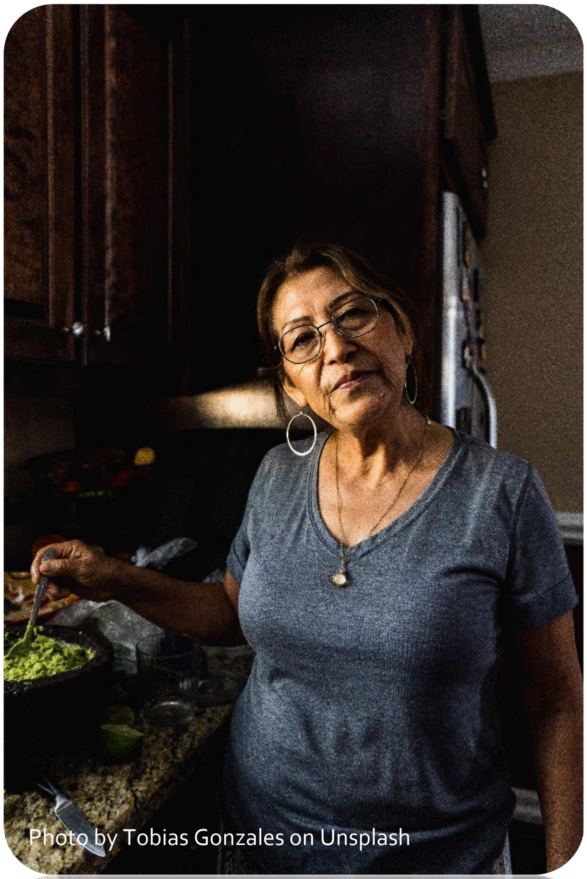
Photo by Tobias Gonzales on Unsplash

Nutrition

The prevalence of obesity in the US has rapidly increased across all races and among Latinx from 1976 to 2003.The rapid increase in obesity is linked with the consumption, availability and promotion of high-calorie, low-nutrient foods[1]. Further, the local food environment (e.g., fast-food outlet versus supermarket density) influences decision making and the ability to adopt a healthy lifestyle[1]. In 2015, only 12.4% of Latinx in Los Angeles County reported having at least 5 servings of fruits/vegetables in the day, and 39% reported drinking at least one soda or sweetened drink per day[5].

Obesity/Overweight

Being overweight and obese are associated with an increased risk for developing many cancers, including those of the breast, colorectum, endometrium, **kidney**, and pancreas, gallbladder cancer and cancers of the **liver,** cervix, and ovary; multiple myeloma; non-Hodgkin lymphoma**;** and aggressive forms of prostate cancer[1]. Over the last 30 years, the prevalence of obesity in the US has rapidly increased across all races and especially among Latinx, **with an alarming two-threefold increase in Latinx children and adolescents**[1]. In Los Angeles, the obesity rate[2] in Latinx is 30.9%, compared to the overall County rate of 25.9%. The rate of overweight Latinx is 39.3%, compared to the County rate of 35.9% [5].

Physical Activity

The rapid increase in obesity is linked with changes in the built environment, including reduced opportunities to be physically active at work or school, while commuting, and during leisure time, resulting in decreased energy expenditure[1]. In Los Angeles, 65.40% of the Latinx community meets the Department of Health and Human Services guidelines for physical activity[3], while 34.60% do not. When looking at **foreign born vs US born Latinx, 40.1% and 26% do not meet the guidelines,** respectively [5, 14].

---

[2] According to NHLBI clinical guidelines, a BMI < 18.5 is underweight, a BMI ≥ 18.5 and < 25 is normal weight, a BMI ≥ 25 and < 30 is overweight, and a BMI ≥ 30 is obese. [REFERENCE: National Heart, Lung, and Blood Institute (NHLBI) http://www.nhlbi.nih.gov/guidelines/obesity/ob_exsum.pdf]

[2]Physical Activity Guidelines for aerobic activity is at least one:1) Vigorous activity for at least 75 minutes/week, 2) Moderate activity for at least 150 minutes/week, or 3) A combination of vigorous and moderate activity for at least 150 minutes/week. http://www.health.gov/paguidelines/pdf/paguide.pdf

Infectious Diseases

Latinx have disproportionately high rates of cancers related to infectious agents, including **liver**, non-Hodgkin lymphoma, and cervical cancer in the US, the **incidence and mortality rates of these cancers are higher among first generation Latinx immigrants** compared to non- Latinx [1, 15]. Infection with Hepatitis B Virus (HBV) or Hepatitis C Virus (HCV) is problematic when the virus becomes chronic. Vaccination against HBV is the best protective measure to reduce prevalence of the virus. For HCV**, deaths associated with the virus are higher among Latinx than non- Latinx**, likely due to less access to screening and treatment for the infection. About 80% of people infected with HCV will become chronically infected and about 65% of people with chronic HCV will develop liver disease, which can lead to liver cancer; the risk of liver disease is higher among heavy alcohol drinkers[1]. Virtually all cervical cancers are caused by persistent Human Papillomavirus (HPV) infection. Increasing uptake in HPV vaccination in adolescents and improving screening and early detection in Latinx women are the primary prevention strategies for reducing HPV associated cancers[1, 10].

About the Cancer Research Center for Health Equity

In 2018, Cedars-Sinai and Samuel Oschin Comprehensive Cancer Institute expanded their research enterprise to include a new center focused on addressing cancer disparities in the community through research, service, and policy. The Cancer Research Center for Health Equity aims to conduct research that is well integrated with community engagement and outreach efforts to reduce cancer incidence/mortality in underserved populations and neighborhoods in Los Angeles County.

References

1. American Cancer Society, *Cancer Facts & Figures for Hispanics/Latinos 2015-2017*. 2017, American Cancer Society: Atlanta.

2. Liu L, W.Y., Sherman RL, Cockburn M, Deapen D,,, *Cancer in Los Angeles County: Trends by Race/Ethnicity, 1976-2012*, in *Los Angeles Cancer Surveillance Program*. 2016, University of Southern California.

3. U.S. Census Bureau. 2011-2015 American Community Survey 5-Year Estimates.

4. Los Angeles County Economic Development Corporation, *An Economic Profile of the Latino Community in Los Angeles County*. 2017: Spotlight on Community Regional Economic Reports.

5. Los Angeles County Department of Public Health. *Los Angeles County Health Survey*. 2018 [cited 2021 February]; Available from: http://www.publichealth.lacounty.gov/ha/hasurveyintro.htm.

6. Pew Research Trends, *Digital Divide Narrows for Latinos as More Spanish Speakers and Immigrants Go Online*, in *Hispanic Trends*. 2016.

7. Pew Research Center, *The Shifting Religious Identity of Latinos in the United States*. 2014.

8. U.S. Census Bureau, *American Community Survey 1-Year Estimates*. 2016.

9. Petrick, J.L., et al., *Future of Hepatocellular Carcinoma Incidence in the United States Forecast Through 2030.* Journal of Clinical Oncology, 2016. **34**(15): p. 1787-+.

10. Islami, F., et al., *Disparities in Liver Cancer Occurrence in the United States by Race/Ethnicity and State.* Ca-a Cancer Journal for Clinicians, 2017. **67**(4): p. 273-289.

11. Sipin, A., Liu, Lihua., Tsai, Kaiya., Deapen, D.,, *Latest Trends in Thyroid Cancer Incidence in Females by Race/Ethnicity in the United States and Los Angeles County*, L.A.C.S. Program, Editor. 2017, University of Southern California Los Angeles, CA.

12. World Cancer Research Fund and American Institute for Cancer Research, *Policy and Action for Cancer Prevention*. 2009: Washington D.C.

13. American Cancer Society, *Cancer Prevention and Early Detection Facts & Figures 2017-2018.* 2017, American Cancer Socity: Atlanta.

14. *California Health Interview Survey, 2015-2016*, UCLA Center for Health Policy Research, Editor.: Los Angeles, CA.

15. Stern, M.C., Fejerman, L., Das, R. et al., *Variability in Cancer Risk and Outcomes Within US Latinos by National Origin and Genetic Ancestry*, in *Current Epidemiology Reports*, C.E. Rep, Editor.

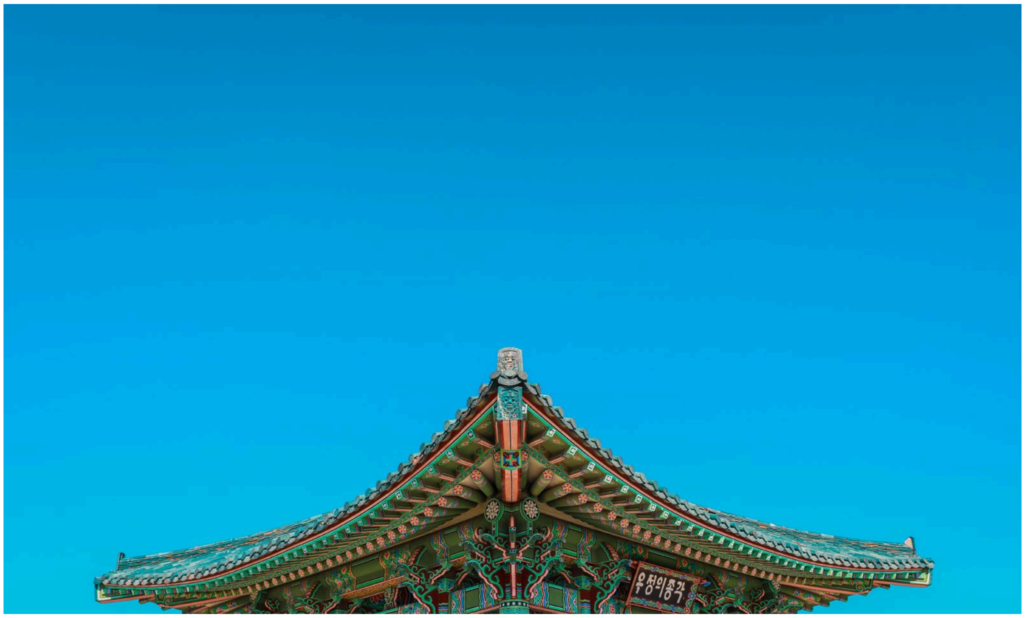

Community Profile: Koreans in Los Angeles County

PUBLISHED BY THE CANCER RESEARCH CENTER FOR HEALTH EQUITY AT CEDARS-SINAI

Photo by Drew Dizzy Graham on Unsplash

Community Overview

Population

Los Angeles County is considered the capital of Asia America, with the largest number of Asian immigrants of any county in the nation. There are a total of 1.4 million Asian Americans in Los Angeles County, which equates to about 14.5% of the county's population[3]. There are approximately 226,000 Korean individuals living in Los Angeles County— making southern California the largest Korean American population in the US. Of those living in the County, 67% are foreign-born [4].

Income

Per capita income among Koreans is $29,590, which is higher than the Los Angeles County average of $28,340. Median household income for Koreans in Los Angeles County is $51,222, compared to the Los Angeles County median of $59,135 [5].

Health Insurance Coverage

Compared to the overall Asian population, Koreans in Los Angeles County have historically had a lower percentage of being insured, however over the past five years that has drastically improved, from 28% uninsured in 2011 to 10.7% uninsured in 2016[4, 5]. The number of Koreans in the County who are utilizing public insurance has increased from 17% to 32.9%, respectively[4, 5]. The overall rate of uninsured in Los Angeles County in 2016 was 9.6%[4].

Sex and Age

Of the Korean population in Los Angeles, roughly 54% are women and 46% are men, with nearly 30% of both genders being over the age of 55. The median age of Korean women and men is 41.9 and 38.3, respectively, compared to the overall County median of 35.8[5].

Poverty and Unemployment

9.3 % of Korean families in Los Angeles County are in poverty, compared to the overall Korean poverty rate of 12.8% in the US[10]. The Los Angeles County unemployment rate for Koreans in the Labor force is 4.4%, compared to the overall LA County rate of 7.5% [5].

Education

92.8% of Koreans ages 25 years and older have at least a high school diploma, compared to 87.7% of Asians and 78.1% of all other residents in Los Angeles County[5].

Mobile Technology

Access to mobile services in South Korea has drastically changed over the past few decades, increasing from 5% in 1990 to 75% in 2001, and as of 2013 over 2/3 of the population owned a smartphone[11, 12]. Despite this trend, Korean elderly living in the US have shown low health literacy and limited credible medical information[13].

FAST FACTS

67%

Of Korean Americans living in Los Angeles County are foreign born[4].

Most Common Cancers

Prostate

Prostate cancer continues to be the most common cancer among most Asian American men. Between 1996-2005, there was a significant increase in incidence among Koreans in the US, increasing 2.9% per year[1]. The risk for prostate cancer has now stabilized, likely due to the wide adoption of the PSA screening test, which is detecting cancer earlier and decreasing mortality[2].

Breast

Breast cancer is generally the most commonly diagnosed cancer in women, varying threefold across Asian American populations. Among Korean women in the US, breast cancer has increased at an alarming rate of 4.7% per year from 1990-2005[1]. The risk for breast cancer among Korean women living in Los Angeles County continues to climb, for both foreign and US born women[2].

Colon and Rectum

There has been a sharp increase in the incidence of colorectal cancer among Koreans in the US, increasing at a rate of 2.2% from 1990-2005[1]. It is the second most commonly diagnosed cancer in both Korean men and women in the US. Nationally, there is a decline in invasive colorectal cancer among Asian Americans largely attributed to higher screening rates, however Korean Americans screening rates are still below the Healthy People 2020 target of 70.5% and the National Colorectal Cancer Roundtable's goal of 80% by 2018[6].

Lung

Lung cancer is the third most common cancer among Koreans in the US. In recent decades, Korean women have experienced an increase in lung cancer[1, 7]. Most cases of lung cancer are caused by smoking cigarettes, for which the prevalence among Asian Americans is not decreasing[1].

Stomach

In both men and women, Koreans in South Korea have the highest stomach cancer incidence rates. In the U.S., Koreans have the highest rate of stomach cancer compared to any other ethnic group. In Angeles County, rates among Korean men remain particularly high, but have stabilized [1, 2, 8].

There is also evidence that Asian Americans have higher rates of liver, cervix, thyroid, and stomach cancers associated with infectious etiologies[1, 9]

Figures 1 & 2

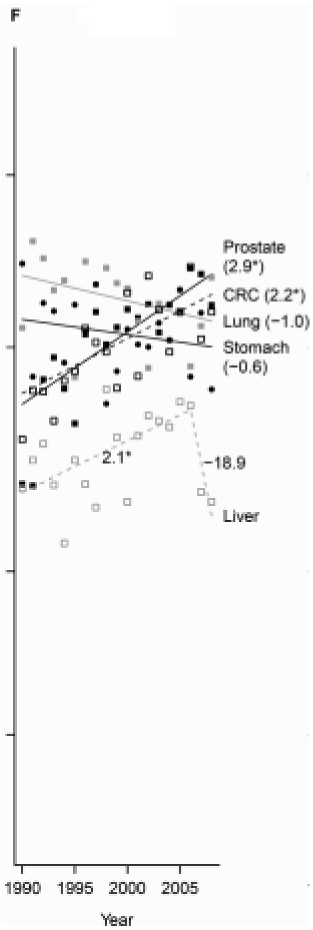

US trends of incidence rates and annual percentage change for the top five cancer sites among **Korean men,** 1990-2008[1].

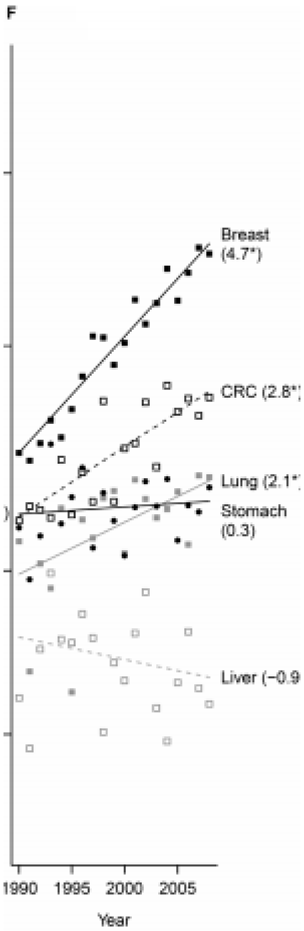

US trends of incidence rates and annual percentage change for the top five cancer sites among **Korean women,** 1990-2008[1].

Figures 1&2 Reprinted from "Cancer Incidence Trends Among Asian American Populations in the United States, 1990-2008". Gomez, S.L., et al., Jnci-Journal of the National Cancer Institute, 2013. 105(15): p. 1096-1110.

Cedars Sinai

Trends in Cancer Type in Koreans

Several cancer incidence trends are on the rise in the Los Angeles Korean community (see Figures 4 & 5). Below are standout trends from 1976 to 2012 by cancer type.

BREAST CANCER

Female breast cancer trends are generally declining or stable among women of almost all races/ethnicities, with exception to Korean women. In particular, the rates of invasive breast cancer among Korean women in Los Angeles County have increased dramatically from 1976-2012 and are now like rates seen in Chinese and South Asian women[2]. Further, proportionally more breast cancers expressed HER2 relative to HR+/HER2– in Korean women, which tends to grow more quickly, spread more aggressively, and present more often as high-grade disease[14].These trends suggest a need for higher rates of mammography screening in these populations. Mammography utilization rates in California are slightly lower in Asian American women than in other racial/ethnic groups. Among Asian Americans, Korean and South Asian women have the lowest mammography utilization, consistent with their higher rates of later-stage disease[1, 14]. Further research should also consider behavioral risk factors, perhaps early-life exposures, and special attention to possible genetic factors.

Colorectal and Thyroid

Caners are now higher among Korean men than Non-Latino white men in Los Angeles County[2, 15].

THYROID CANCER

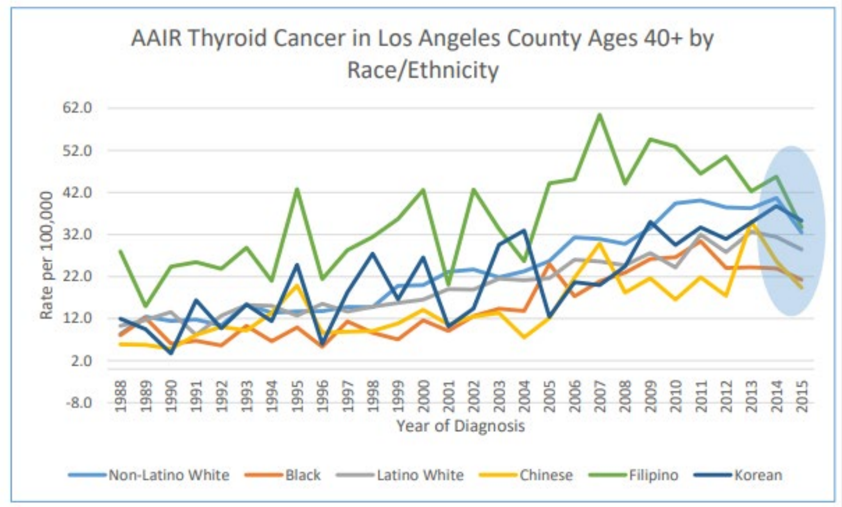

Thyroid cancer is now the most common type of cancer diagnosed in South Korea, with more than 40,000 people in the country diagnosed with the disease in 2011[16]. In Los Angeles County, there is a sustained increase in thyroid cancer rates among Korean women aged 40 years and above, see Figure 3[15]. There are unique epidemiologic patterns by cell type, sex (female), and age which suggest the increasing trends may be due to a combination of enhanced diagnostic procedures as well as an actual increase in etiologic risk[2].

Note: Figure 3 reprinted from "Latest Trends in Thyroid Cancer Incidence in Females by Race/Ethnicity in the United States and Los Angeles County", Sipin, A., Liu, Lihua., Tsai, Kaiya., Deapen, D.,, L.A.C.S. Program, 2017, University of Southern California Los Angeles, CA.

PROSTATE CANCER

Incidence of prostate cancer has dramatically changed over the course of four decades in Los Angeles County. Prostate cancer has been increasing in Korean men, with a delayed peak after the adoption of the PSA test. There was a small decrease in incidence after the peak; however, the rate is now about the same it was before the PSA test introduction[2]. Although some of the trend may be attributed to improved screening, rising incidence rates have been noted in other Asian countries where the screening is not as common. Associations are being drawn with changing lifestyle factors such as a heavier protein and fat dietary pattern with decreased consumption of phytochemicals common in a traditional Asian diet[1].

COLORECTAL CANCER

Cancers of the colon and rectum combined are the third most commonly diagnosed cancers among both men and women in Los Angeles County, with incidence on the rise among Koreans[2]. Korean men have now surpassed Non- Latino white men in incidence of Colorectal Cancer, 54.7 and 51.0 per 100,000, respectively[2]. Historically, Koreans have shown very low screening utilization, with even lower utilization among uninsured Koreans in Los Angeles County, although this appears to be improving across California in the past decade[6, 17]. In addition, health behaviors such as poor nutrition and a westernized diet consisting of high proportions of meat and processed meat, meat by-products, fast foods, and sweets; lack of physical activity; smoking; and alcohol consumption are linked to higher prevalence of colorectal cancer, [1, 18].

Figures 4 & 5

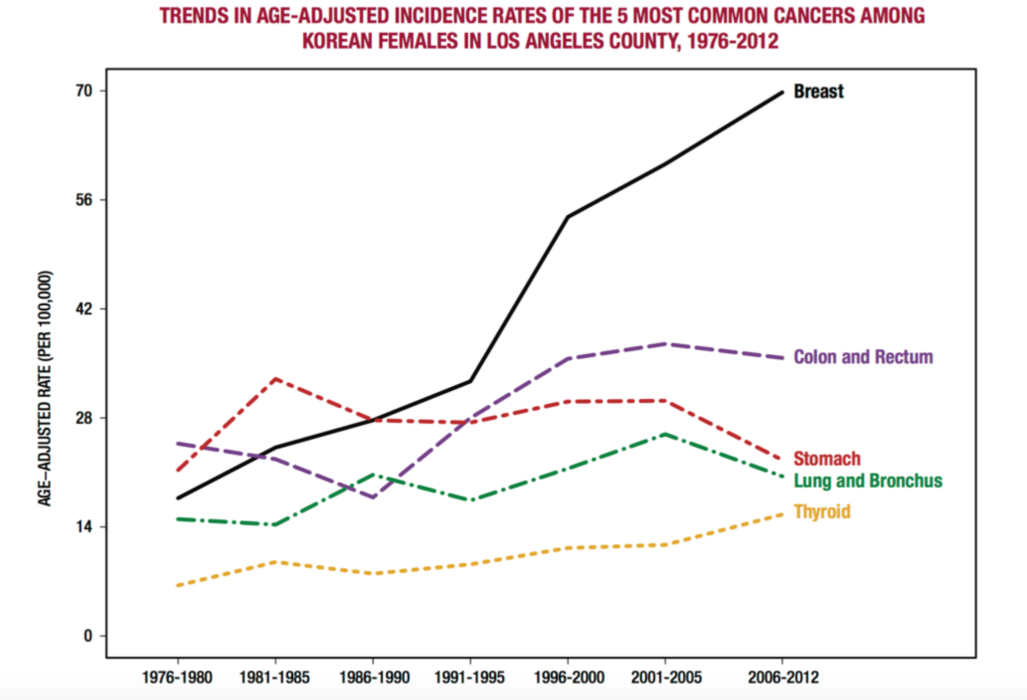

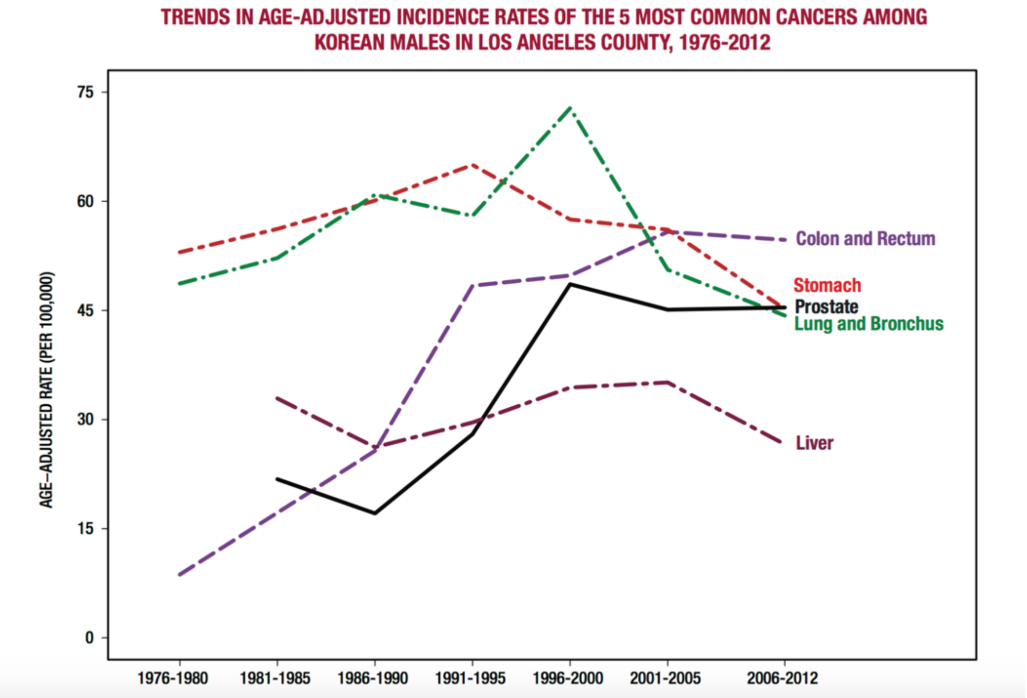

Note. Trends in Incidence Rates Among Korean Males and Females. Reprinted from "Cancer in Los Angeles County: Trends by Race/Ethnicity, 1976-*2012*," by Liu L, W.Y., Sherman RL, Cockburn M, Deapen D. in *Los Angeles Cancer Surveillance Program*. 2016, University of Southern California. Reprinted with permission.

Risk Behaviors

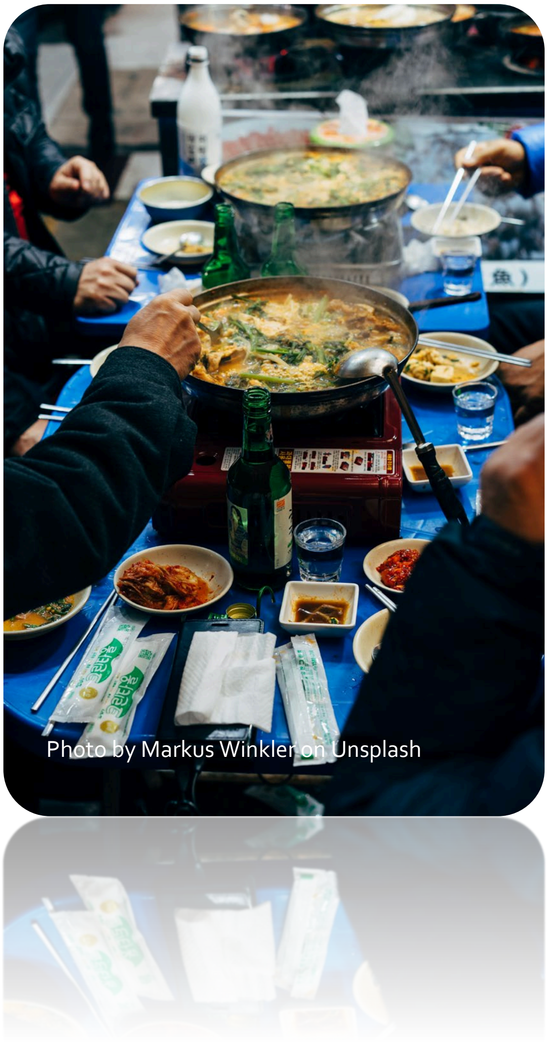
Photo by Markus Winkler on Unsplash

Disparities among Asian American groups have become more pronounced, **with greater incidence among those who are foreign-born, lower socio-economic status, and living in areas with high ethnic concentration**[1]. This finding highlights the need for life-saving prevention and early detection measures that are key to reducing the cancer burden in the Korean community. Organized efforts to reduce tobacco use, improve diet, and increase the use of established screening tests can save lives [19]. It is estimated that 20% of all cancers diagnosed in the US are caused by a combination of excess body weight, physical inactivity, excess alcohol consumption, and poor nutrition[20, 21].

Nutrition

Although Koreans in California have a low prevalence of obesity (2.1% in 2012), there is still good evidence to suggest that risk for **colon and rectum cancer, prostate, and breast cancer** is increased with poor nutrition [20-23]. The International Agency for Research on Cancer (IARC) recently classified processed meat (lunch meat, bacon hot dog) as a human carcinogen and red meat (beef, lamb, pork) as a likely carcinogen based on their association with increased **colorectal cancer** risk. A recent study looking at dietary patterns and colorectal cancer in Koreans found that a westernized diet, consisting of high proportions of meat and processed meat, meat by-products, fast foods, and sweets, showed a positive association with **colorectal cancer risk**, especially among women (OR = 2.13)[18]. A diet high in calories, fat, and red meat may also increase risk for **prostate cancer**, as can too little intake of calcium and plant foods rich in vitamin B and fiber[1, 21]. There is strong evidence on the benefit of vegetable and fruit consumption on cancer risk, with a diet of low meat/starches and a high intake of vegetables and legumes associated with a reduced risk of **breast cancer** in Asian Americans [21, 23].

Cancer Screening

Mammography rates among Asian Americans continues to lag behind rates in the general population[1]. With the incidence of **breast cancer** rising in Korean women, earlier detection of breast cancer can lead to increased 5-year survival rates, and a greater range of and less invasive treatment options[14, 21]. Asian Americans are also less likely to be screened for **colorectal cancer** compared with non-Hispanic Whites, with historically wider disparities for Koreans[6]. When looking at underserved Korean Americans in Los Angeles County, screening rates for **colorectal cancer** have been very low, although that appears to be changing[17]. A recent California based study found that between 2003 and 2009, colorectal cancer screening prevalence increased from 43.3% to 64.6% in Asian Americans and from 58.1% to 71.4% in Non-Hispanic Whites; a subgroup analysis of Koreans showed a 94% increase in colorectal screening during this time[6]. Despite these improvements, screening among Koreans remains below the Healthy People 2020 target of 70.5% and the National Colorectal Cancer Roundtable's goal of 80% by 2018. Colorectal cancer is often characterized as the most preventable, but least prevented cancer[17, 24]. When colorectal cancer is diagnosed at the localized stage, five-year survival is 90%;- still only 39% of cases in the US are diagnosed at this stage[21].

Smoking

The incidence pattern of lung cancer by nativity is consistent with the prevalence of smoking among Korean men; however, among women, the prevalence of smoking is higher among U.S.-born with 41% having reported ever smoking[1], compared to 15 % of foreign-born women, which is counter to their incidence patterns[7]. In addition to lung cancer, **smoking increases the risk of colorectal, breast, advanced stage prostate**, and liver cancer, among several others[21]. Three in ten cancer deaths in the US are associated with smoking, and although that number is decreasing, smoking prevalence among Asian Americans is not[1, 21]. Smokers who quit can expect to gain as many as 10 years of life expectancy. Further, smokers who are diagnosed with cancer are more likely to quit than those not diagnosed and they have better health outcomes[21]. Culturally tailored tobacco cessation interventions are still needed among Asian Americans, and particularly Koreans.

Infectious Diseases

Incidence of liver cancer is increasing for all Asian Americans in Los Angeles County, including Koreans, with greater disparities among first generation Korean immigrants[1, 2]. There is an association with Hepatitis C Virus (HCV) and liver cancer, as nearly 80% of people infected with HCV will become chronically infected and about 65% of people with chronic HCV will develop liver disease, which can lead to liver cancer; the risk being highest among heavy alcohol drinkers[20]. There is no vaccine to protect against HCV infection, so prevention efforts must include education for at-risk groups about exposure and information for infected individuals about transmission[21]. Koreans have historically had very high level of Hepatitis B Virus (HBV) prevalence, which has decreased with changing health behaviors, less transmission and importantly the HBV vaccine[25]. Screening rates for cervical cancer have been increasing among Koreans in California (from 68% in 2003 to 71% in 2007) and infections associated with *Helicobacter pylori (H. pylori)* which is highest among Korean immigrants, is on the decline[2, 21].

Alcohol

In Korea, the proportion of deaths due to alcohol is estimated at 8.9%, far exceeding the global estimate of 3.8%[26]. When looking at ethnic drinking cultures in the U.S., Koreans have higher alcohol consumption rates compared to other Asian American groups[27]. In 2015, 35.9% of Korean adults in Los Angeles County reported binge drinking[2][28]. Alcohol consumption is an established risk factor for cancers of the mouth, pharynx, larynx, esophagus, liver, **colorectum,** and **female breast**, and may increase the risk of pancreatic cancer[21] . Given that Koreans engage in more moderate- and high-risk drinking, campaigns to limit alcohol consumption according to American Cancer Society's nutrition and physical activity guidelines for cancer prevention and risk reduction[3] should be implemented.

About the Cancer Research Center for Health Equity

In 2018, Cedars-Sinai and Samuel Oschin Comprehensive Cancer Institute expanded their research enterprise to include a new center focused on addressing cancer disparities in the community through research, service, and policy. The Cancer Research Center for Health Equity aims to conduct research that is well integrated with community engagement and outreach efforts to reduce cancer incidence/mortality in underserved populations and neighborhoods in Los Angeles County.

[1] Ever smoker defined as having smoked at least 100 cigarettes over lifetime

[2] Males are considered binge drinkers if they consumed 5 or more alcoholic drinks on at least one occasion in the past year. Females are considered binge drinkers if they consumed 4 or more alcoholic drinks on at least one occasion in the past year.

[3] no more than two drinks per day for men and no more than one drink per day for women

References

1. Gomez, S.L., et al., *Cancer Incidence Trends Among Asian American Populations in the United States, 1990-2008.* Jnci-Journal of the National Cancer Institute, 2013. **105**(15): p. 1096-1110.

2. Liu L, W.Y., Sherman RL, Cockburn M, Deapen D,,, *Cancer in Los Angeles County: Trends by Race/Ethnicity, 1976-2012*, in *Los Angeles Cancer Surveillance Program*. 2016, University of Southern California.

3. Los Angeles County Economic Development Corporation, *An Economic Profile of the Asian Community in Los Angeles County*. 2017: Spotlight on Community Economic Reports.

4. U.S. Census Bureau, *American Community Survey 1-Year Estimates*. 2016.

5. U.S. Census Bureau. 2011-2015 American Community Survey 5-Year Estimates.

6. Fedewa, S.A., et al., *Temporal Trends in Colorectal Cancer Screening among Asian Americans.* Cancer Epidemiol Biomarkers Prev, 2016. **25**(6): p. 995-1000.

7. Raz, D.J., et al., *Epidemiology of Non-small Cell Lung Cancer in Asian Americans Incidence Patterns Among Six Subgroups by Nativity.* Journal of Thoracic Oncology, 2008. **3**(12): p. 1391-1397.

8. Kim, Y., et al., *Stomach cancer incidence rates among Americans, Asian Americans and Native Asians from 1988 to 2011.* Epidemiol Health, 2015. **37**: p. e2015006.

9. Islami, F., et al., *Disparities in Liver Cancer Occurrence in the United States by Race/Ethnicity and State.* Ca-a Cancer Journal for Clinicians, 2017. **67**(4): p. 273-289.

10. Pew Research Center analysis of 2013-2015 American Community Survey (IPUMS).

11. Yoo, Y., K. Lyytinen, and H.D. Yang, *The role of standards in innovation and diffusion of broadband mobile services: The case of South Korea.* Journal of Strategic Information Systems, 2005. **14**(3): p. 323-353.

12. Shin, S., *A Comparative Study Of Smartphone Addiction Drivers' Effect On Work Performance In The U.S. And Korea.* The Journal of Applied Business Research 2016. **32**(2).

13. Oh, Y.S., E.Y. Choi, and Y.S. Kim, *Predictors of Smartphone Uses for Health Information Seeking in the Korean Elderly.* Soc Work Public Health, 2018. **33**(1): p. 43-54.

14. Gomez, S.L., et al., *Breast cancer in Asian Americans in California, 1988-2013: increasing incidence trends and recent data on breast cancer subtypes.* Breast Cancer Res Treat, 2017. **164**(1): p. 139-147.

15. Sipin, A., Liu, Lihua., Tsai, Kaiya., Deapen, D.,, *Latest Trends in Thyroid Cancer Incidence in Females by Race/Ethnicity in the United States and Los Angeles County*, L.A.C.S. Program, Editor. 2017, University of Southern California Los Angeles, CA.

16. Ahn, H.S., H.J. Kim, and H.G. Welch, *Korea's thyroid-cancer "epidemic"--screening and overdiagnosis.* N Engl J Med, 2014. **371**(19): p. 1765-7.

17. Jo, A.M., et al., *Colorectal cancer screening among underserved Korean Americans in Los Angeles County.* J Immigr Minor Health, 2008. **10**(2): p. 119-26.

18. Park, Y., et al., *Dietary patterns and colorectal cancer risk in a Korean population: A case-control study.* Medicine (Baltimore), 2016. **95**(25): p. e3759.

19. World Cancer Research Fund and American Institute for Cancer Research, *Policy and Action for Cancer Prevention*. 2009: Washington D.C.

20. American Cancer Society, *Cancer Facts & Figures for Hispanics/Latinos 2015-2017*. 2017, American Cancer Society: Atlanta.

21. American Cancer Society, *Cancer Prevention and Early Detection Facts & Figures 2017-2018.* 2017, American Cancer Socity: Atlanta.

22. Wolstein, J., Babey SH., Diamant, AL., *Obesity in California*. 2015, UCLA Center for Health Policy Research: Los Angeles, CA.

23. Wu, A.H., et al., *Dietary patterns and breast cancer risk in Asian American women.* Am J Clin Nutr, 2009. **89**(4): p. 1145-54.

24. Itzkowitz, S.H., *Incremental advances in excremental cancer detection tests.* J Natl Cancer Inst, 2009. **101**(18): p. 1225-7.

25. Hyun, C.S., et al., *Chronic hepatitis B in Korean Americans: decreased prevalence and poor linkage to care.* BMC Infect Dis, 2016. **16**(1): p. 415.

26. Ryu, S.Y., C.M. Crespi, and A.E. Maxwell, *Drinking patterns among Korean adults: results of the 2009 Korean community health survey.* J Prev Med Public Health, 2013. **46**(4): p. 183-91.

27. Cook, W.K., N. Mulia, and K. Karriker-Jaffe, *Ethnic drinking cultures and alcohol use among Asian American adults: findings from a national survey.* Alcohol Alcohol, 2012. **47**(3): p. 340-8.

28. *California Health Interview Survey, 2015-2016*, UCLA Center for Health Policy Research, Editor.: Los Angeles, CA.

Cedars Sinai

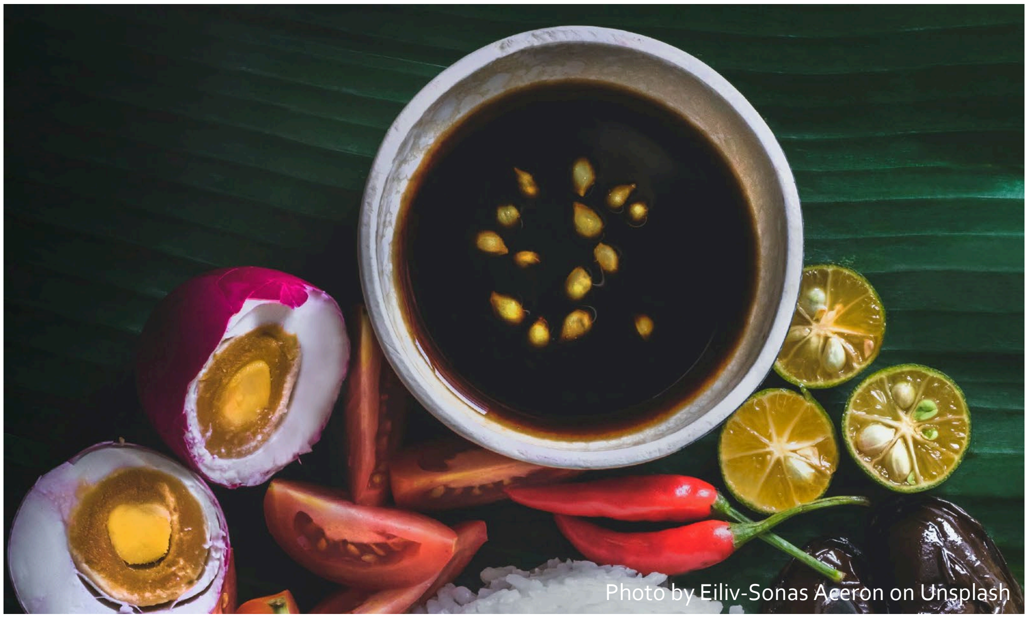
Photo by Eiliv-Sonas Aceron on Unsplash

Community Profile: Filipinos in Los Angeles County

PUBLISHED BY THE CANCER RESEARCH CENTER FOR HEALTH EQUITY AT CEDARS-SINAI

Community Overview

Population

Los Angeles County is considered the capital of Asia America, with the largest number of Asian immigrants of any county in the nation. There are a total of 1.4 million Asian Americans in Los Angeles County, which equates to about 14.5% of the county's population. Filipinos are the second-largest Asian American group in Los Angeles. There are approximately 395,580 Filipino individuals living in the County—60% of whom are foreign-born[3, 4].

Income

Per capita income among Filipino individuals is $31.273, which is higher than the Los Angeles County average of $28,340. The median household income for Filipinos in Los Angeles County is $85.289, compared to the Los Angeles County median of $59,135[4].

Health Insurance Coverage

In 2016, 88.4% of Filipinos in Los Angeles had health insurance coverage while 11.6% were uninsured. In 2016 the overall County rate of uninsured was 9.6%[4, 6].

Poverty and Unemployment

4.6 % of Filipino families in Los Angeles County are in poverty, compared to the overall Filipino poverty rate in the US of 5.1%, and the County poverty rate for families at 13.9%[4, 6]. The Los Angeles County unemployment rate for Filipinos in the Labor force is 3.7%, compared to the overall LA County rate of 7.5%[4] .

Occupation

Historically, Filipinos have comprised a large proportion of the health workforce and many Filipino immigrants who arrived in the US in the 1970s and 1980s came over with expertise in the health field. Up until the mid-1980s, Filipino nurses represented approximately 75% of all foreign nurses in the US nurse workforce[9].

Education

94.5% of Filipinos ages 25 years and older have at least a high school diploma, compared to 87.7% of all Asian Americans and 78.1% of all other residents in Los Angeles County[4]. In 2016, one study found that Filipino Americans had the second largest proportion of college graduates among Asian Americans in the County, with 76.2% having at least a bachelor's degree[10].

Sex and Age

Of the Filipino population in Los Angeles, roughly 53% are women and 47% are men, with the median age being 39.5-- compared to the overall County median age of 35.8[4].

Residency

Filipino Americans in Los Angeles have often been referred to as having "residential invisibility'. In 2002, The City of Los Angeles designated a section of Westlake as Historic Filipino-town; however, this area is now largely populated by Latino Americans. Although about 25% of Filipinos still live in Filipino town, many live in adjacent communities like Westlake, Koreatown, East Hollywood, Silver Lake, and Echo Park. Other large concentrations of Filipinos can be found in suburbs like Carson, West Covina, Hacienda Heights, Rowland Heights, and Walnut[11].

Religion

About eight-in-ten Filipinos (81%) identify as Catholic; while a smaller number of Filipino Americans (65%) identify as Catholic, most Filipinos retain their Catholic spiritual beliefs and practices upon immigration[12]. More research shows that Filipino Americans' high level of religiosity impacts how they view health and illness and that Catholic churches are a trusted, and often preferred source for support and health information[13].

FAST FACTS

60%

Of Filipino Americans living in Los Angeles County are foreign born.

Most Common Cancers

Breast

Breast cancer is generally the most commonly diagnosed cancer among Asian American women, with some of the highest rates among Filipinas[1]. Rates have increased for distant-stage disease among Filipinas in the US (2.2% per year) and for Filipina women under the age of 50.Compared to Non-Hispanic Whites, Filipinas have higher incidence rates of some HER2+ subtypes[1, 2].

Prostate

Prostate cancer is the most commonly diagnosed cancer among Filipino American men, accounting for 29.4% of all cancers cases[3]. Filipino men also saw a significant annual increase in prostate cancer between 1990-1993 (19%) but the risk for prostate cancer has now stabilized, likely due to the wide adoption of the PSA screening test, which is detecting cancer earlier and decreasing mortality[1, 3].

Lung and Bronchus

Lung cancer remains the leading cause of cancer death in the US, and Lung and Bronchus cancer are the second most common cancer among Filipino men and third among Filipina women[1, 3].Filipina Americans experienced statistically significant increases in lung cancer (2.1% per year) from 1990-2008[1]. Rates are decreasing for lung cancers of squamous cell and increasing for adenocarcinoma, which is now the most common histologic type of tumor in both women and men[3].

Colon and Rectum (CRC)

CRC is the second most common cancer among Filipina women and third among Filipino men[1, 3]. Incidence rates have not significantly varied among Filipinos in past decades, however late-stage diagnosis and poor survival prognosis is increasing among Filipinos[5]. Filipino Americans screening rates are still below the Healthy People 2020 target of 70.5% and the National Colorectal Cancer Roundtable's goal of 80% by 2018[7].

Uterus

Uterine cancer, often referred to as endometrial cancer, is predominantly found among non-Latina whites; however, there has been a substantial increase in rates among Filipinas, which is now approaching that of non-Latina whites[1, 3]. Migrant studies showed that US-born Asians (including Filipinas) had higher endometrial cancer incidence than their Asian-born counterparts, suggesting that environmental exposures in Asian Americans may be a contributing factor[8].

Figures 1 & 2

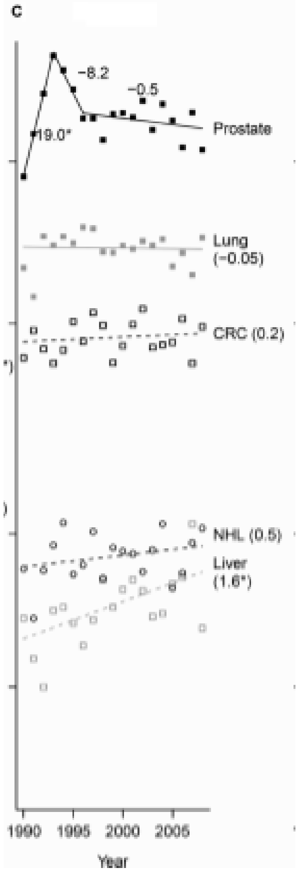

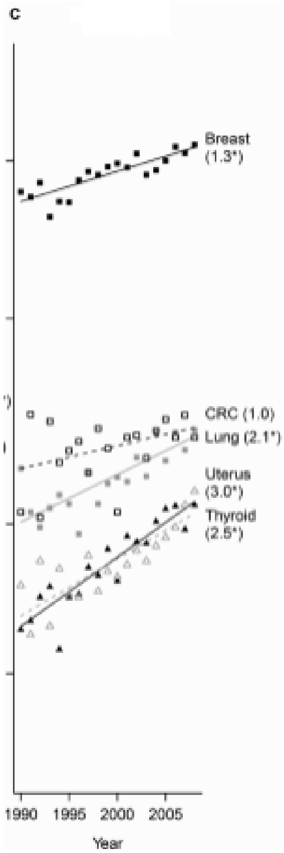

US trends of incidence rates and annual percentage change for the top five cancer sites among **Filipino men,** 1990-2008.

US trends of incidence rates and annual percentage change for the top five cancer sites among **Filipino women,** 1990-2008.

Figures 1&2 Reprinted from "Cancer Incidence Trends Among Asian American Populations in the United States, 1990-2008". Gomez, S.L., et al., Jnci-Journal of the National Cancer Institute, 2013. 105(15): p. 1096-1110.

Trends in Cancer Type in Filipinos

Several cancer incidence trends are on the rise in the Los Angeles Filipino community (see Figures 4 & 5). Below are standout trends from 1976 to 2012 by cancer type.

BREAST CANCER

Among Filipina women in the US and in the Philippines, rates of breast cancer have been steadily increasing over the past three decades[1, 3]. Rates among Filipina Americans under age 50 are now comparable to those in non-Hispanic White (NHW) women. Filipina women also experience proportionally more breast cancers expressing HER2 relative to HR+/HER2–, which tends to grow more quickly, spread more aggressively, and present more often as high-grade disease[2]. Trends in incidence of invasive breast cancer among Filipinas, who have adopted more of a U.S. lifestyle, are parallel to those among non-Latina whites. These trends suggest a need for higher rates of mammography screening in these populations. Mammography utilization rates in California are slightly lower in Asian American women than in other racial/ethnic groups (e.g., 62%-68% in Filipinas receiving a mammogram within the past 2 years, relative to 72.4% in the overall US population) and still well below the Healthy People 2020 target[1, 2].

Thyroid Cancer

Filipinos have the highest incidence rate of thyroid cancer among all racial/ethic groups in Los Angeles County[3].

UTERINE CANCER

Incidence rates of uterine cancer among Filipina American women has risen 3% per year from 1990-2008[1]. In the Philippines, rates were lower but still increased over the same time-period. In the 1990s, Los Angeles County began to see a substantial increase in rate of uterine cancer among Filipinas, which is now approaching that of non-Latina whites[3]. Uterine cancer is believed to be caused by fluctuation in the balance of hormones in women (estrogen and progesterone). Pregnancy, increasing number of births, and oral contraceptives (birth control pills) that contain estrogen and progesterone are believed to be protective factors against uterine cancer[3]. Obesity is also a major risk factor in uterine cancer as fat cells are a major source of estrogen[3, 14].

THYROID CANCER

Filipinos in the US have seen a significant increase in thyroid cancer, with an average increase of 2.5% from 1990-2005; in Los Angeles County, Filipinos have the highest incidence rates among all racial/ethnic groups[1, 3]. Filipinos also have a higher rate of thyroid cancer recurrence and mortality. In one study that looked at age adjusted mortality rates due to thyroid cancer in the US, rates were highest in Filipinos (1.72 deaths per 100,000 population) compared with all other Asian Americans (1.03 per 100,000 population) and Non-Hispanic Whites (1.17 per 100,000 population); being highly educated was also associated with particularly high proportionate mortality compared with all other groups[9]. The reasons behind this increase in thyroid incidence and mortality among Filipinos is still unclear, however theories around cultural factors, such as diet, or environmental exposures, exposure to ionizing radiation, or genetic factors remain of interest[9].

KIDNEY CANCER

In Los Angeles County, incidence of kidney cancer has been increasing in Filipino men. Cigarette smoking is an important cause of kidney cancer, and smokers who quit tobacco see a significant decrease in risk[3]. Other risk factors are obesity, hypertension, and having certain inherited conditions, including von Hippel-Lindau disease, BirtHogg-Dube syndrome, tuberous sclerosis, and familial papillary renal cell carcinoma. However, the increase in cancer risk has been argued to at least partially be attributable to improved diagnosis, as incidence rates of late stage kidney cancers in Los Angeles, like the rest of the U.S., have been fairly stable [3].

LIVER CANCER

Filipino men experienced a statistically significant increase in liver cancer in the US from 1990-2008, at a rate of 1.6% each year[1]. When looking at Los Angeles County, Asian Americans tend to have the highest age-adjusted incidence rates, and men in particular, including Filipino men[3]. The increasing prevalence of obesity and diabetes in Los Angeles County, and Hepatitis B and Hepatitis C are the strongest risk factors for liver cancer. Some have estimated that nearly 36% of liver cancer in the US is attributed to obesity and diabetes[15, 16].

Figures 4 & 5

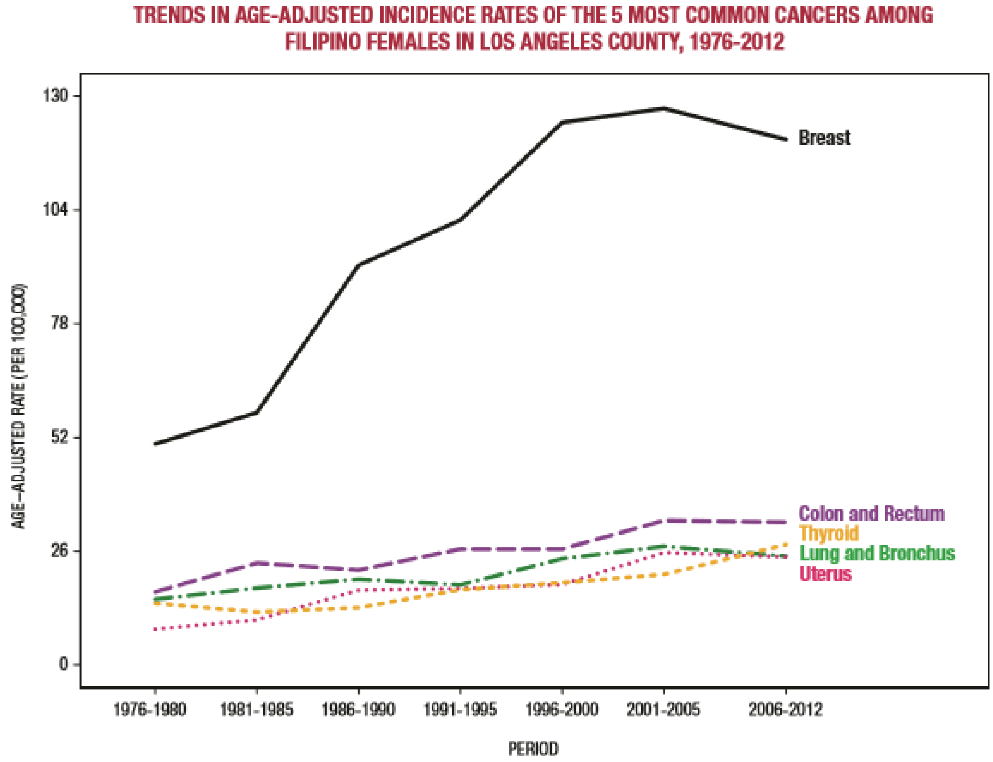

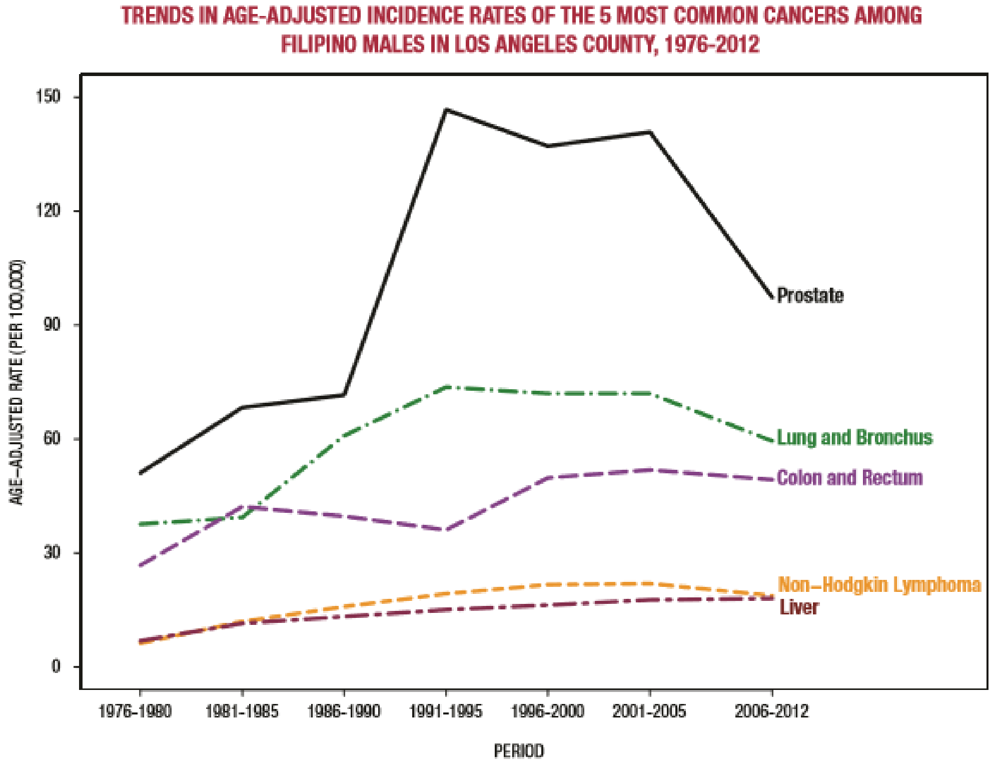

Note. Trends in Incidence Rates Among Korean Males and Females. Figures 4&5 Reprinted from "Cancer in Los Angeles County: Trends by Race/Ethnicity, 1976-2012," by Liu L, W.Y., Sherman RL, Cockburn M, Deapen D. in *Los Angeles Cancer Surveillance Program*. 2016, University of Southern California. Reprinted with permission.

Risk Behaviors

Disparities among Asian Americans are becoming more pronounced. There are opportunities for life-saving prevention and early detection measures that are key to reducing the cancer burden in the Filipino community. Organized efforts to reduce tobacco use, improve diet, maintain healthy weight, and increase the use of established screening tests can save lives. It is estimated that 20% of all cancers diagnosed in the US are caused by a combination of excess body weight, physical inactivity, excess alcohol consumption, and poor nutrition.

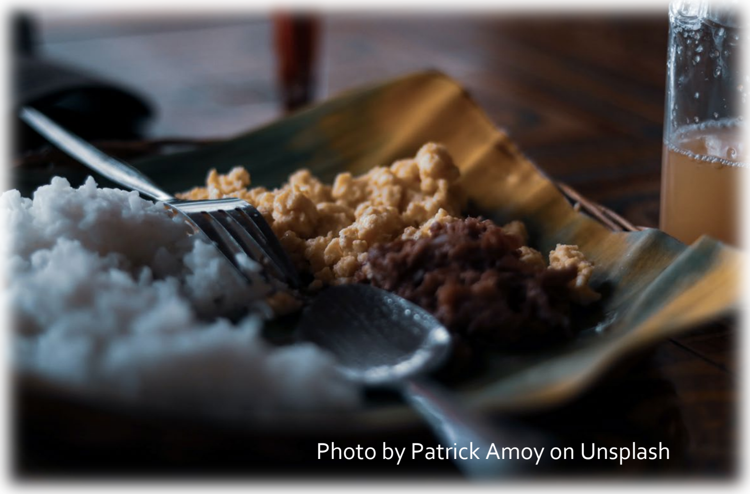

Photo by Patrick Amoy on Unsplash

Nutrition and Obesity

Filipina Americans have **the highest average body mass index** (BMI) of all Asian American ethnicities, with a sharp increase in the prevalence of obesity in the past 20 years[14]. Filipino immigrants have also seen the most dramatic increase in rates of obesity due to adoption of the westernized diet, consisting of high proportions of meat and processed meat, meat by-products, fast foods, and sweets. In 2016, 72.1% of Filipinos in California self-reported a BMI that put them at either increased risk or higher high risk[1] of obesity[17]. There is sufficient evidence to conclude that being overweight or obese increases the risk of developing 13 different cancers: **uterine**, esophagus (adenocarcinoma), **liver,** stomach (gastric cardia), **kidney** (renal cell), brain (meningioma), multiple myeloma, pancreas, **colorectum**, gallbladder, ovary, **breast (postmenopausal)**, and **thyroid**[15, 18, 19]. There is also mounting evidence suggesting that obesity increases the risk of cancer recurrence and second primary tumors, and decreases survival for several cancers[15]. A diet high in calories, fat, and red meat may also increase risk for **prostate cancer**, as can too little intake of calcium and plant foods rich in vitamin B and fiber[1]. There is increasing information on the benefit of vegetable and fruit consumption on cancer risk, with a diet of low meat/starches and a high intake of vegetables and legumes associated with a reduced risk of **breast cancer** in Asian Americans[15, 19].

Cancer Screening

Life-saving screening tests for **colorectal cancer** have not been well utilized among Filipino Americans, resulting in late stage of diagnosis and poor survival relative to other racial/ethnic groups[5]. Community surveys have found that Filipino Americans are significantly less likely than other racial/ethnic groups to receive a diagnosis of localized or Stage I disease and that Filipino American males have poorer 5-year survival after colorectal cancer than the other racial/ethnic groups (56% versus 63% among Whites)[5]. Screening among Filipinos remains below the Healthy People 2020 target of 70.5% and the National Colorectal Cancer Roundtable's goal of 80% by 2018. Colorectal cancer is often characterized as the most preventable, but least prevented cancer. When colorectal cancer is diagnosed at the localized stage, five-year survival is 90%; still only 39% of cases in the US are diagnosed at this stage[15, 20].

Increasing rates of **breast cancer** among young Filipina women and the increasing trends of late-stage disease suggests the need for better mammography screening in these populations. In California, mammography rates have been historically lower in Asian American women, including Filipinas, than in NHW, Blacks, and Hispanics[2].

Smoking

Although Filipino men have a consistent pattern of lung cancer with rates of smoking, Filipina women have a higher prevalence of smoking among those who are U.S.-born with 24% having reported ever smoking[2], compared to 15 % of foreign-born women, which is counter to their incidence patterns[21]. In addition to lung cancer, **smoking likely increases the risk of colorectal, breast, advanced stage prostate, and liver cancer,** among several others. Three in ten cancer deaths in

[1] Body Mass Index: WHO Definition - 4 level (adult only) cutoffs: 18.5 - 22.99 (Increasing but acceptable risk), 23.0 - 27.49 (Increased risk), 27.5 or higher (Higher high risk)
[2] Ever smoker defined as having smoked at least 100 cigarettes over lifetime

the US are associated with smoking, and although that number is decreasing, smoking prevalence among Asian Americans is not. Smokers who quit can expect to gain as many as 10 years of life expectancy. Further, smokers who are diagnosed with cancer are more likely to quit than those not diagnosed, and they have better health outcomes than cancer cases who continue to smoke.

Infectious Diseases

Filipinos living in Los Angeles County have experienced an increase in **liver cancer[1, 3]**. There is an association with Hepatitis C Virus (HCV) and liver cancer, as nearly 80% of people infected with HCV will become chronically infected and about 65% of people with chronic HCV will develop liver disease, which can lead to liver cancer; the risk being highest among heavy alcohol drinkers[22]. There is no vaccine to protect against HCV infection, so prevention efforts must include education for at-risk groups about exposure and information for infected individuals about transmission[15].

Chronic infection of hepatitis B virus (HBV) can also cause **liver cancer**[15]. The Philippines Department of Health and multiple research studies have shown a high rate of chronic hepatitis B infection in the Philippines, not only in high-risk populations but in the general population[23]. With so many foreign-born Filipinos living in the US, effective approaches to vaccination uptake and screening must be established, with special attention to the adult population of health-care workers who are at increased risk of workplace-acquired HBV infection[15].

About the Cancer Research Center for Health Equity

In 2018, Cedars-Sinai and Samuel Oschin Comprehensive Cancer Institute expanded their research enterprise to include a new center focused on addressing cancer disparities in the community through research, service, and policy. The Cancer Research Center for Health Equity aims to conduct research that is well integrated with community engagement and outreach efforts to reduce cancer incidence/mortality in underserved populations and neighborhoods in Los Angeles County.

References

1. Gomez, S.L., et al., *Cancer Incidence Trends Among Asian American Populations in the United States, 1990-2008.* Jnci-Journal of the National Cancer Institute, 2013. **105**(15): p. 1096-1110.

2. Gomez, S.L., et al., *Breast cancer in Asian Americans in California, 1988-2013: increasing incidence trends and recent data on breast cancer subtypes.* Breast Cancer Res Treat, 2017. **164**(1): p. 139-147.

3. Liu L, W.Y., Sherman RL, Cockburn M, Deapen D,,, *Cancer in Los Angeles County: Trends by Race/Ethnicity, 1976-2012*, in *Los Angeles Cancer Surveillance Program*. 2016, University of Southern California.

4. U.S. Census Bureau, *American Community Survey 1-Year Estimates*. 2016.

5. Maxwell, A.E., L.L. Danao, and R. Bastani, *Dissemination of colorectal cancer screening by Filipino American community health advisors: a feasibility study.* Health Promot Pract, 2013. **14**(4): p. 498-505.

6. U.S. Census Bureau. 2011-2015 American Community Survey 5-Year Estimates.

7. Fedewa, S.A., et al., *Temporal Trends in Colorectal Cancer Screening among Asian Americans.* Cancer Epidemiol Biomarkers Prev, 2016. **25**(6): p. 995-1000.

8. Setiawan, V.W., *Endometrial Cancer Among Asian Americans*. Cancer Epidemiology Among Asian Americans, ed. S.D.e. Wu A. 2016, Springer, Cham.

9. Nguyen, M.T., et al., *Thyroid cancer mortality is higher in Filipinos in the United States: An analysis using national mortality records from 2003 through 2012.* Cancer, 2017. **123**(24): p. 4860-4867.

10. Melany De La Cruz-Viesca; Zhenxiang Chen; Paul M. Ong; Darrick Hamilton; William A. Darity Jr., *The Color of Wealth in Los Angeles*. 2016, A Joint Publication of Duke University, The New School, the University of California, Los Angeles and the Insight Center for Community Economic Development.

11. Trinidad, E., *L.A.'s Historic Filipinotown Turns Ten: What's Changed?*, KCET, Editor. 2012.

12. Pew Research Center, *Asian Americans: A Mosaic of Faiths*, in *The Pew Forum on Religion & Public Life*. 2012.

13. Lagman, R.A., et al., *"Leaving it to God" religion and spirituality among Filipina immigrant breast cancer survivors.* J Relig Health, 2014. **53**(2): p. 449-60.

14. *Cancer epidemiology in Asian Americans*. 2016, New York, NY: Springer Science+Business Media. pages cm.

15. American Cancer Society, *Cancer Prevention and Early Detection Facts & Figures 2017-2018.* 2017, American Cancer Socity: Atlanta.

16. Islami, F., et al., *Disparities in Liver Cancer Occurrence in the United States by Race/Ethnicity and State.* Ca-a Cancer Journal for Clinicians, 2017. **67**(4): p. 273-289.

17. *California Health Interview Survey, 2015-2016*, UCLA Center for Health Policy Research, Editor.: Los Angeles, CA.

18. Wolstein, J., Babey SH., Diamant, AL., *Obesity in California*. 2015, UCLA Center for Health Policy Research: Los Angeles, CA.

19. Wu, A.H., et al., *Dietary patterns and breast cancer risk in Asian American women.* Am J Clin Nutr, 2009. **89**(4): p. 1145-54.

20. Itzkowitz, S.H., *Incremental advances in excremental cancer detection tests.* J Natl Cancer Inst, 2009. **101**(18): p. 1225-7.

21. Raz, D.J., et al., *Epidemiology of Non-small Cell Lung Cancer in Asian Americans Incidence Patterns Among Six Subgroups by Nativity.* Journal of Thoracic Oncology, 2008. **3**(12): p. 1391-1397.

22. American Cancer Society, *Cancer Facts & Figures for Hispanics/Latinos 2015-2017*. 2017, American Cancer Society: Atlanta.

23. Gish, R.G., et al., *Chronic hepatitis B virus in the Philippines.* J Gastroenterol Hepatol, 2016. **31**(5): p. 945-52.

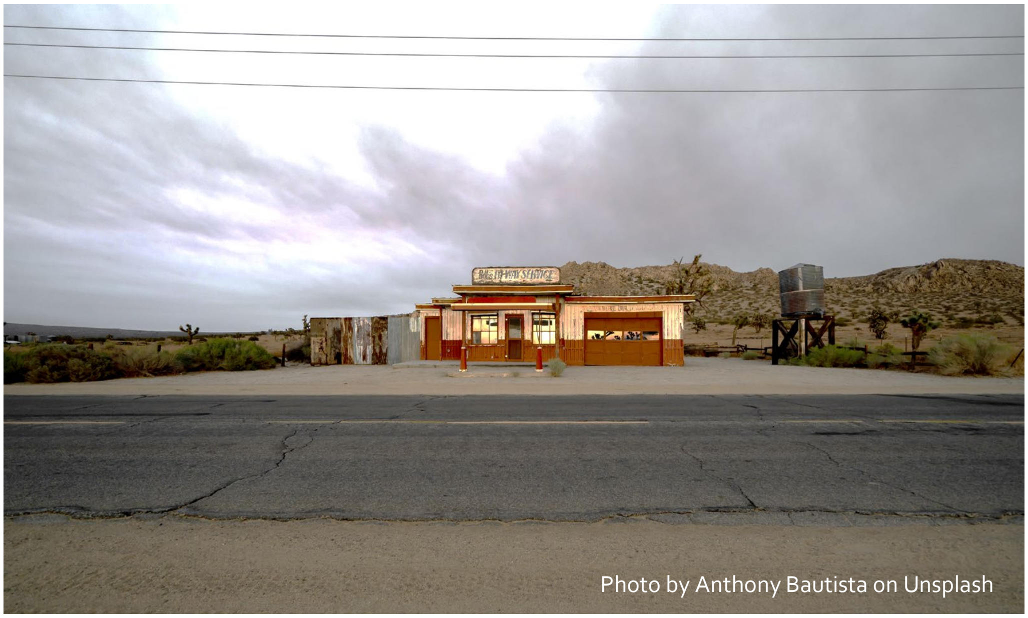
Photo by Anthony Bautista on Unsplash

Community Profile: Antelope Valley (SPA1)

PUBLISHED BY THE CANCER RESEARCH CENTER FOR HEALTH EQUITY AT CEDARS-SINAI

Community Overview

Population

Antelope Valley (AV), or Service Planning Area (SPA) 1, is in northern Los Angeles County (LAC) and constitutes the western tip of the Mojave Desert. There are approximately 390,938 people living in this rural suburb, of whom 45% are Latino, 35% White, 16% Black, 4% Asian, .4% American Indian, .2% Native Hawaiian/Pacific Islander[3]. AV has the second-highest African American population in the County.

Mental Health

AV has a higher number of adults ever diagnosed with depression compared to the overall County rate, 15%, and 12% respectively. In 2014, it was reported that only 8% of adults accessed mental health care[3]. In focus groups conducted by the Kaiser Hospital Foundation, community members emphasized the lack of available mental health services in the AV, especially for children[4]. Barriers to accessing mental health care in the AV include a severe shortage of providers, lack of resources in the community, poor quality of care, and lack of sensitivity to racial/ethnic minority groups[4]. Further, poverty, cost of living, and immigration status were identified as the top contributors to mental illness in the community by mental health providers[4].

Access to Care

There is a shortage of adequate health care and health information in the AV. Lack of adequate health providers, low visibility of resources, cost of insurance and medication, transportation, appointment availability, lack of culturally competent care, lack of choice in procedures, and difficulty navigating the medical system all contribute to the regions glaring health disparities. This inequitable access to care is often the most reported barrier to health and wellbeing from residents in this region[4].

Economic Insecurity

The high cost of housing, high cost of food, and low educational attainment contributes to economic insecurity in the AV. This affects residents' ability to maintain a healthy lifestyle and prevent and manage chronic health conditions. Many residents have to commute multiple hours a day to make a livable wage, therefore they do not have time to cook or exercise[4]. Further, students reported cost (48%) and other financial reasons (54%) among the top three barriers to educational attainment.

Life Span

Residents living in the AV are in the 32nd percentile for health opportunities among all California residents with approximately 202,964 people living in severely under-resourced census tracts. Essentially, this means that nearly 50% to 70% of Californians have a greater opportunity to live a long healthy life than residents living in this region[4].

FAST FACTS

African Americans in SPA1 die **four years sooner** than black residents elsewhere in the county and nearly **ten years earlier** than county residents in general[5].

Physical Environment

In the County, AV has the lowest percentage (40%) of adults who use walking paths, parks, playgrounds, or sports fields in their neighborhood and the highest percentage of adults (24%) who live in neighborhoods where there are no walking paths, parks, playgrounds, or sports fields in their neighborhood. Further, it has the greatest percentage of adults (26%) with over an hour commute to work, compared to 12% in LAC[3].

Cancer Disparities

Incidence

The Antelope Valley has higher rates of breast cancer (21%), lung cancer (12%), and colorectal cancer (16%), compared to the overall population in the County [Figure 1]. Uterine/cervical cancer numbers are less available due to the lack of access to gynecologic oncology in the valley, forcing residents to travel to Los Angeles or San Bernardino to access care[1].

Mortality

More people die from lung, breast, and colorectal in the Antelope Valley than any other region in Los Angeles County[Figure 2][2]

Figure 1

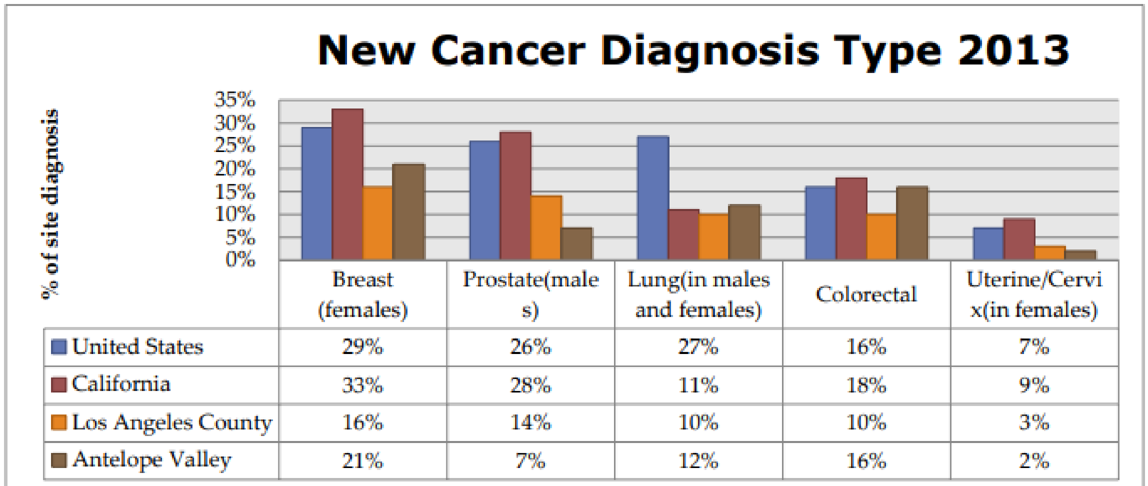

	Breast (females)	Prostate(males)	Lung(in males and females)	Colorectal	Uterine/Cervix(in females)
United States	29%	26%	27%	16%	7%
California	33%	28%	11%	18%	9%
Los Angeles County	16%	14%	10%	10%	3%
Antelope Valley	21%	7%	12%	16%	2%

Data Source: (American Cancer Society, 2013) (Antelope Valley Hospital Cancer Registry , 2013)

Figure 1: Reprinted from "Antelope Valley Hospital Needs Assessment Oncology Report" by Paez, M., Karnstedt, P., 2015.

Figure 2

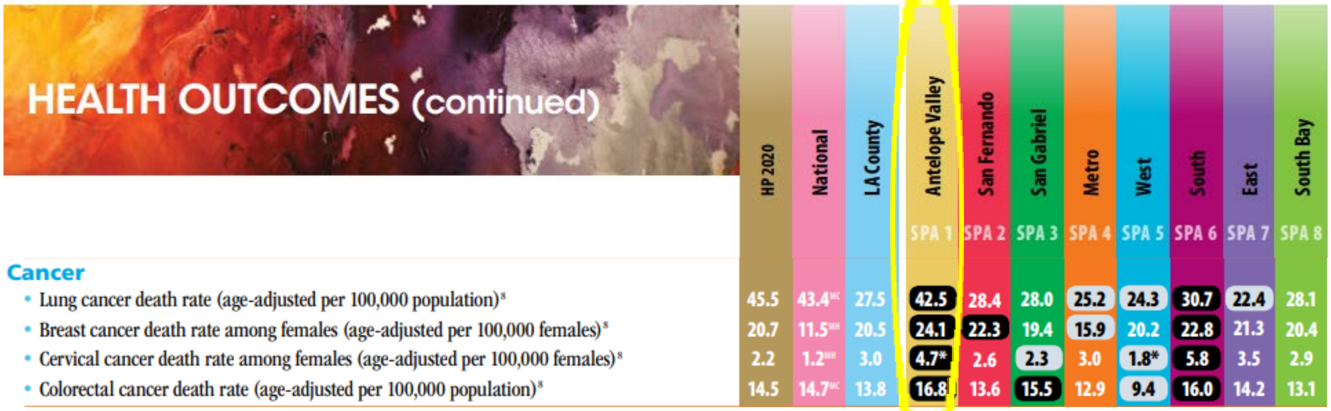

Cancer	HP 2020	National	LA County	Antelope Valley SPA 1	San Fernando SPA 2	San Gabriel SPA 3	Metro SPA 4	West SPA 5	South SPA 6	East SPA 7	South Bay SPA 8
• Lung cancer death rate (age-adjusted per 100,000 population)[8]	45.5	43.4[MC]	27.5	42.5	28.4	28.0	25.2	24.3	30.7	22.4	28.1
• Breast cancer death rate among females (age-adjusted per 100,000 females)[8]	20.7	11.5[MH]	20.5	24.1	22.3	19.4	15.9	20.2	22.8	21.3	20.4
• Cervical cancer death rate among females (age-adjusted per 100,000 females)[8]	2.2	1.2[MH]	3.0	4.7*	2.6	2.3	3.0	1.8*	5.8	3.5	2.9
• Colorectal cancer death rate (age-adjusted per 100,000 population)[8]	14.5	14.7[MC]	13.8	16.8	13.6	15.5	12.9	9.4	16.0	14.2	13.1

Figure 2: Reprinted from "Key Indicators of Health by Service Planning Area" by Los Angeles Cou[illegible] Department of Public Health, 2017.

Risk Behaviors

Antelope Valley residents of all races face higher mortality rates than in the rest of the County, as well as a unique set of disparities that are often present in more rural communities. There are opportunities for life-saving prevention and early detection measures that are key to reducing the cancer burden in this region. Organized efforts to reduce tobacco use, improve diet, maintain healthy weight, and increase the use of established screening tests can save lives. It is estimated that 20% of all cancers diagnosed in the US are caused by a combination of excess body weight, physical inactivity, excess alcohol consumption, and poor nutrition.

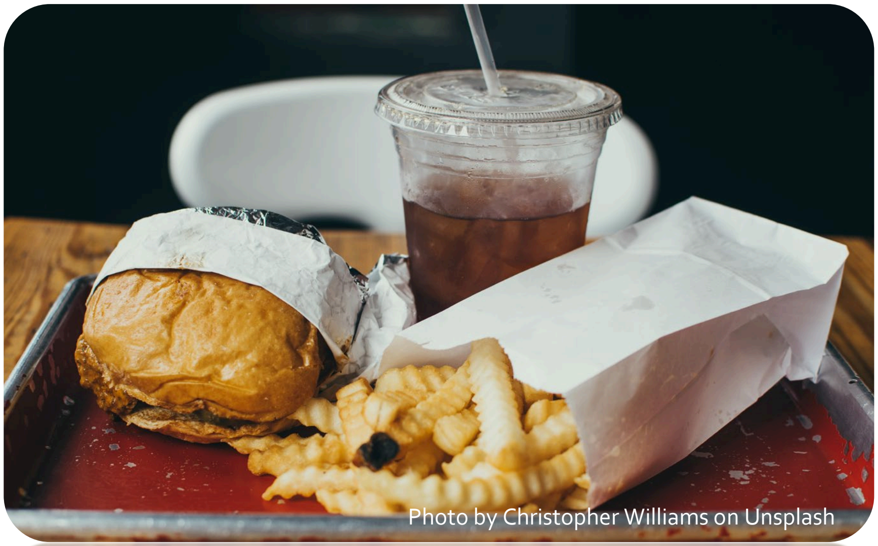

Photo by Christopher Williams on Unsplash

Obesity

AV has the highest percentage of adults who are obese (35%) and the highest number of adults who consume at least one soda or sweetened beverage per day (58%) in the County. 61% of children consume fast food at least once per week and 38% of children consume at least one soda or sugary beverage per day[3]. Reported barriers to care for obesity in the AV include: cost of healthy food and gyms, availability of fresh food, and transportation issues[4].There is sufficient evidence to conclude that being overweight or obese increases the risk of developing 13 different cancers: **uterine**, esophagus (adenocarcinoma), **liver,** stomach (gastric cardia), **kidney** (renal cell), brain (meningioma), multiple myeloma, pancreas**, colorectum**, gallbladder, ovary, **breast (postmenopausal**), and **thyroid**[6-8]. There is also mounting evidence suggesting that obesity increases the risk of cancer recurrence and second primary tumors, and decreases survival for several cancers[8]. A diet high in calories, fat, and red meat may also increase risk for **prostate cancer**, as can too little intake of calcium and plant foods rich in vitamin B and fiber[9].

Cancer Screening

Life-saving cancer screening tests are not widely available in the AV, and residents often must travel several hours to access these services. **Cancer screening programs and education are urgently needed in the community** to address the cancer incidence and mortality disparities in the region.

Although AV is doing better than Los Angeles County overall when it comes to colorectal cancer screening (63%; 95% CI: 55.5-70.4%), its rate of compliance is still below the Healthy People 2030 target[10,11].

AV lags behind the rest of the County in the percentage of women who reported to have a pap smear within the past 3 years (76.7%; 95% CI: 68.4-85.0%), and who reported to have a mammogram within the past 2 years (74.4%; 95% CI: 66.0-82.9%)[10].

Smoking

AV has the greatest number of adults (16%) who smoke cigarettes compared to any other SPA in Los Angeles County[3]. In addition to lung cancer, **smoking increases the risk of colorectal, breast, advanced stage prostate, and liver cancer**, among several others. Three in ten cancer deaths in the US are associated with smoking and smokers who quit can expect to gain as many as 10 years of life expectancy. Further, smokers who are diagnosed with cancer are more likely to quit than those not diagnosed, and they have better health outcomes.

Spotlight on African Americans

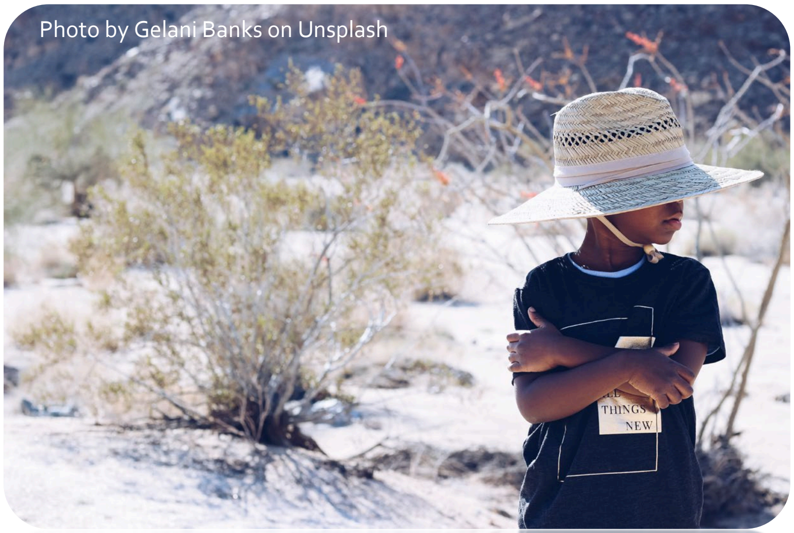

Cities like Palmdale and Lancaster in the AV have been a growing home to African Americans over the past several decades and are considered a racial enclave for black residents in the County. However, life is shorter for African Americans in the AV, and disparities among black residents are even more pronounced. African Americans in SPA1 die four years sooner than black residents elsewhere in the county and nearly 10 years earlier than county residents in general[5].

Health Disparities

Infant mortality is highest among African Americans who live in the Antelope Valley, compared to other black residents in the County, due to of a lack of prenatal care and access to health providers and clinics. In addition to mortality, black residents in the AV are obese at above average rates (31%), have ahigh rates of cardiovascular disease (28%) and high rates of stroke at 40%, when compared to other ethnic and racial groups in the County[4,5]. Lastly, blacks in the AV have a lower median household income and a higher percentage of the population that lives in poverty compared to other racial/ethnic groups in SPA1 [Figure 3][3].

Figure 3

Health Indicator	Los Angeles County	SPA 1				
		SPA 1 Overall	White	Black	Latino	Asian
% of adults with less than a high school diploma[3]	24%	22%	9%	13%	42%	10%
% of population that lives in poverty (household income <100% Federal Poverty Level [FPL])[3]	17%	19%	12%	32%	22%	11%[a]
Median household income[3]	$56,241	$57,423	$68,498	$43,034	$49,352	$76,263
% of households that are crowded[b,3]	12%	6%	2%	5%	12%	4%
% of population that is foreign-born[3]	35%	18%	5%	3%[a]	30%	60%
Life expectancy (in years)[4]	81.8	78.0	76.4	73.8	83.0	85.2[c]

Figure 3: Reprinted from "Supplement to Community Health Assessment, Service Planning Area 1: Antelope Valley" by Los Angeles County Department of Public Health, 2014.

Tobacco Use

It is well known that African Americans have the highest smoking rates among all racial/ethnic groups in the US. The tobacco use rate for African Americans living in Californian is 20%, compared to the 13.5% rate of use among non-Hispanic white (NHW) Californians[12]. When looking at low-income African Americans in California, the adult smoking rate is 26.9%, and among African Americans who have not completed high school, rates are at a staggering 40.2%[13]. In LAC, African Americans are more likely to smoke than any other racial/ethnic group (25% versus 15% NHW, 12% Latino, 11% API)[14], and we would expect to see the same statewide trend of higher rates among low-income and under-educated blacks in this specific region. Further, menthol cigarettes are disproportionately smoked by the African American population; in a large population-based sample of US residents aged 12 and older, 89.9% of African Americans smoked mentholated cigarettes, compared to 25.7% of NHWs[15,16]. Despite smoking fewer cigarettes per day, African American cessation rates are the lowest among all racial ethnic groups (3.3% compared to 6% in non-Hispanic Whites)[17,18]. In multiple study populations, smokers of menthol cigarettes were less likely to quit successfully than smokers of regular cigarettes[17,19-23]. Inversely, interest in quitting is higher in African Americans compared to non-Hispanic Whites (75.6% compared to 69.1%)[18]. The high preference for menthol cigarettes may contribute to the excess smoking-related morbidity experienced by African Americans. Further, if smokers of menthol cigarettes have lower cessation rates and thereby have longer duration of smoking compared to smokers of nonmentholated cigarettes, this could contribute to increased health risks and morbidly [17].

Second-hand Smoke

African American children are the most likely to live with someone who smokes; in fact, African American children are more than three times as likely as any other racial/ethnic group to live in a home where smoking is permitted[10]. Exposure to second-hand smoke is the third leading cause of preventable death in the U.S. The most serious type of exposure to children occurs: 1) before birth via maternal smoking, and 2) childhood exposure by parental smoking. The United States Environmental Protection Agency estimates that second-hand smoke causes more than 300,000 cases of asthma, bronchitis, middle ear infections, and pneumonia in children each year. **African American smokers and their children living in the AV are among one of the highest risk groups for cancer mortality in all of Los Angeles County, presenting an opportunity for much needed tobacco cessation, early detection, and cancer prevention interventions.**

Conclusion

There is a critical gap in access to care in the Antelope Valley, particularly among the African American population whose outcomes in this region are vastly inequitable. The AV is a region that is often overlooked and understudied in the County, and we do not clearly understand the factors contributing to high mortality, especially among black residents. Social and built environment factors such as level of education and jobs; health behaviors, including smoking, food access, and obesity; walkability and green space in the community; and access and quality of healthcare should be examined as potential indicators for health equity interventions.

About the Cancer Research Center for Health Equity

In 2018, Cedars-Sinai and Samuel Oschin Comprehensive Cancer Institute expanded their research enterprise to include a new center focused on addressing cancer disparities in the community through research, service, and policy. The Cancer Research Center for Health Equity aims to conduct research that is well integrated with community engagement and outreach efforts to reduce cancer incidence/mortality in underserved populations and neighborhoods in Los Angeles County.

References

1. Paez M, Karnstedt, P. *Needs Assessment Report (Oncology)*. 2015.

2. Los Angeles County Department of Public Health. *Key Indicators of Health by Service Planning Area*. 2017. http://publichealth.lacounty.gov/ha/docs/2015LACHS/KeyIndicator/PH-KIH_2017-sec%20UPDATED.pdf

3. Los Angeles County Department of Public Health. *Supplement to Community Health Assessment, Service Planning Area 1: Antelope Valley*. 2014.

4. Kaiser Foundation Hospital. *2019*. Community Health Needs Assessment. *Kaiser Permanente Southern California Region Community Benefit CHNA Report for KFH-Panorama City*.

5. ANN M. SIMMONS. For Antelope Valley African Americans, a lower life expectancy *Los Angeles Times*. https://www.latimes.com/local/la-xpm-2012-may-29-la-me-av-black-health-20120529-story.html

6. Wolstein J, Babey SH., Diamant, AL. *Obesity in California*. 2015. http://healthpolicy.ucla.edu/publications/Documents/PDF/2015/obesityreport-jun2015.pdf

7. Wu AH, Yu MC, Tseng CC, Stanczyk FZ, Pike MC. Dietary patterns and breast cancer risk in Asian American women. *Am J Clin Nutr*. Apr 2009;89(4):1145-54. doi:10.3945/ajcn.2008.26915

8. American Cancer Society. *Cancer Prevention and Early Detection Facts & Figures 2017-2018*. 2017.

9. Gomez SL, Noone AM, Lichtensztajn DY, et al. Cancer Incidence Trends Among Asian American Populations in the United States, 1990-2008. *Jnci-J Natl Cancer I*. Aug 2013;105(15):1096-1110. doi:10.1093/jnci/djt157

10. Los Angeles County Department of Public Health. Los Angeles County Health Survey. Accessed February, 2021. http://www.publichealth.lacounty.gov/ha/hasurveyintro.htm

11. Office of Disease Prevention and Health Promotion. Healthy People 2030. 2021. https://health.gov/healthypeople/objectives-and-data/browse-objectives/cancer

12. UCLA Center for Health Policy Research. California Health Interview Survey.

13. Data from: California Health Interview Survey, 2015-2016. *Los Angeles, CA*. Deposited December 2017.

14. County of Los Angeles Public Health. *Cigarette Smoking in Los Angeles County: Local Data to Inform Tobacco Policy*. 2010. *A Cities and Community Health Report*.

15. Caraballo RS, Asman K. Epidemiology of menthol cigarette use in the United States. *Tob Induc Dis*. May 23 2011;9 Suppl 1:S1. doi:10.1186/1617-9625-9-S1-S1

16. Giovino GA, Villanti AC, Mowery PD, et al. Differential trends in cigarette smoking in the USA: is menthol slowing progress? *Tob Control*. Jan 2015;24(1):28-37. doi:10.1136/tobaccocontrol-2013-051159

17. Okuyemi KS, Ebersole-Robinson M, Nazir N, Ahluwalia JS. African-American menthol and nonmenthol smokers: differences in smoking and cessation experiences. *J Natl Med Assoc*. Sep 2004;96(9):1208-11.

18. Centers for Disease Control and Prevention. *Quitting smoking among adults--United States, 2001-2010*. Vol. 60. 2011:1513-1519.

19. Delnevo CD, Gundersen DA, Hrywna M, Echeverria SE, Steinberg MB. Smoking-cessation prevalence among U.S. smokers of menthol versus non-menthol cigarettes. *Am J Prev Med*. Oct 2011;41(4):357-65. doi:10.1016/j.amepre.2011.06.039

20. Gandhi KK, Foulds J, Steinberg MB, Lu SE, Williams JM. Lower quit rates among African American and Latino menthol cigarette smokers at a tobacco treatment clinic. *Int J Clin Pract*. Mar 2009;63(3):360-7. doi:10.1111/j.1742-1241.2008.01969.x

21. Levy DT, Blackman K, Tauras J, et al. Quit attempts and quit rates among menthol and nonmenthol smokers in the United States. *Am J Public Health*. Jul 2011;101(7):1241-7. doi:10.2105/AJPH.2011.300178

22. Lewis M, Wang Y, Berg CJ. Tobacco control environment in the United States and individual consumer characteristics in relation to continued smoking: differential responses among

menthol smokers? *Prev Med*. Aug 2014;65:47-51. doi:10.1016/j.ypmed.2014.04.019

23. Trinidad DR, Perez-Stable EJ, Messer K, White MM, Pierce JP. Menthol cigarettes and smoking cessation among racial/ethnic groups in the United States. *Addiction*. Dec 2010;105 Suppl 1:84-94. doi:10.1111/j.1360-0443.2010.03187.x
